# 31st Annual Meeting and Associated Programs of the Society for Immunotherapy of Cancer (SITC 2016): late breaking abstracts: National Harbor, MD, USA. 9-13 November 2016

**DOI:** 10.1186/s40425-016-0191-4

**Published:** 2016-12-08

**Authors:** Sonja Althammer, Keith Steele, Marlon Rebelatto, Tze Heng Tan, Tobias Wiestler, Guenter Schmidt, Brandon Higgs, Xia Li, Li Shi, Xiaoping Jin, Joyce Antal, Ashok Gupta, Koustubh Ranade, Gerd Binning, Joaquim Bellmunt, Ronald de Wit, David J. Vaughn, Yves Fradet, Jae Lyun Lee, Lawrence Fong, Nicholas J. Vogelzang, Miguel A. Climent, Daniel P. Petrylak, Toni K. Choueiri, Andrea Necchi, Winald Gerritsen, Howard Gurney, David I. Quinn, Stéphane Culine, Cora N. Sternberg, Yabing Mai, Markus Puhlmann, Rodolfo F. Perini, Dean F. Bajorin, Padmanee Sharma, Margaret K. Callahan, Emiliano Calvo, Joseph W. Kim, Filipo de Braud, Patrick A. Ott, Petri Bono, Rathi N. Pillai, Michael Morse, Dung T. Le, Matthew Taylor, Pavlina Spilliopoulou, Johanna Bendell, Dirk Jaeger, Emily Chan, Scott J. Antonia, Paolo A. Ascierto, Delphine Hennicken, Marina Tschaika, Alex Azrilevich, Jonathan Rosenberg, Ofer Levy, Christopher Chan, Gady Cojocaru, Spencer Liang, Eran Ophir, Sudipto Ganguly, Amir Toporik, Maya Kotturi, Tal Fridman Kfir, Benjamin M. Murter, Kathryn Logronio, Liat Dassa, Ling Leung, Shirley Greenwald, Meir Azulay, Sandeep Kumar, Zoya Alteber, Xiaoyu Pan, Arthur Machlenkin, Yair Benita, Andrew W. Drake, Ayelet Chajut, Ran Salomon, Ilan Vankin, Einav Safyon, John Hunter, Zurit Levine, Mark White, Rom Leidner, Hyunseok Kang, Robert Haddad, Neil H. Segal, Lori J. Wirth, Robert L. Ferris, F. Stephen Hodi, Rachel E. Sanborn, Thomas F. Gajewski, William Sharfman, Dan McDonald, Shivani Srivastava, Xuemin Gu, Penny Phillips, Chaitali Passey, Tanguy Seiwert, Tsadik Habtetsion, Gang Zhou, Donastas Sakellariou-Thompson, Cara Haymaker, Caitlin Creasy, Mark Hurd, Naohiro Uraoka, Jaime Rodriguez Canales, Scott Koptez, Patrick Hwu, Anirban Maitra, Chantale Bernatchez, Scott M. Coyle, Kole T. Roybel, Levi J. Rupp, Stephen P. Santoro, Stephanie Secrest, Michael Spelman, Hanson Ho, Tina Gomes, Tiffany Tse, Chia Yung-Wu, Jack Taunton, Wendell Lim, Peter Emtage, Tarsem Moudgil, Carmen Ballesteros-Merino, Traci Hilton, Christopher Paustian, Rom Leidner, David Page, Walter Urba, Bernard Fox, Bryan Bell, Ashish Patel, Tove Olafsen, Daulet Satpayev, Michael Torgov, Filippo Marchioni, Jason Romero, Ziyue Karen Jiang, Charles Zamilpa, Jennifer S. Keppler, Alessandro Mascioni, Fang Jia, Chen-Yu Lee, Jean Gudas, Ryan J. Sullivan, Yujin Hoshida, Theodore Logan, Nikhil Khushalani, Anita Giobbie-Hurder, Kim Margolin, Joanna Roder, Rupal Bhatt, Henry Koon, Thomas Olencki, Thomas Hutson, Brendan Curti, Shauna Blackmon, James W. Mier, Igor Puzanov, Heinrich Roder, John Stewart, Asim Amin, Marc S. Ernstoff, Joseph I. Clark, Michael B. Atkins, Howard L. Kaufman, Jeffrey Sosman, Sabina Signoretti, David F. McDermott, Abraham A. Anderson, Igor Puzanov, Mohammed M. Milhem, Robert H. I. Andtbacka, David Minor, Kevin S. Gorski, Daniel M. Baker, Omid Hamid, Howard L. Kaufman, Emmanuel Akporiaye, Brendan Curti, Yoshinobu Koguchi, Rom Leidner, Kim Sutcliffe, Kristie Conder, Walter Urba, Thomas Marron, Nina Bhardwaj, Linda Hammerich, Fiby George, Seunghee Kim-Schulze, Tibor Keler, Tom Davis, Elizabeth Crowley, Andres Salazar, Joshua Brody, Arta Monjazeb, Megan E. Daly, Jonathan Riess, Tianhong Li, William J. Murphy, Karen Kelly, Zhiwei Hu, Rulong Shen, Amanda Campbell, Elizabeth McMichael, Lianbo Yu, Bhuvaneswari Ramaswam, Cheryl A. London, Tian Xu, William Carson, Kathleen M. Kokolus, Elizabeth A. Repasky, Todd D. Schell, Joseph D. Drabick, David J. Messenheimer, Shawn Jensen, Bernard Fox, Mark Rubinstein, Kristina Andrijauskaite, Marzena Swiderska-syn, Kristin Lind, Agnes Choppin, Marina K. Roell, John Wrangle, Kristina Andrijauskaite, Marzena Swiderska-syn, Peter Rhode, Hing Wong, Mark Rubinstein, Shamim Ahmad, Mason Webb, Rasha Abu-Eid, Rajeev Shrimali, Vivek Verma, Atbin Doroodchi, Zuzana Berrong, David Yashar, Raed Samara, Mikayel Mkrtichyan, Samir Khleif, Steven Powell, Mark Gitau, Christopher Sumey, Andrew Terrell, Michele Lohr, Ryan K. Nowak, Steven McGraw, Ash Jensen, Miran Blanchard, Kathryn A. Gold, Ezra E. W. Cohen, Christie Ellison, Lora Black, John Lee, William Chad Spanos, Erik Wennerberg, Emily Schwitzer, Claire Lhuillier, Graeme Koelwyn, Rebecca Hiner, Lee Jones, Sandra Demaria, Vandeveer Amanda, John W. Greiner, Jeffrey Schlom, Michelle Bookstaver, Christopher M. Jewell, Christopher Paustian, Andrew Gunderson, Brian Boulmay, Rui Li, Bradley Spieler, Kyle Happel, Tarsem Moudgil, Zipei Feng, Carmen Ballesteros-Merino, Christopher Dubay, Brenda Fisher, Yoshinobu Koguchi, Sandra Aung, Eileen Mederos, Carlo B. Bifulco, Michael McNamara, Keith Bahjat, William Redmond, Augusto Ochoa, Hong-Ming Hu, Adi Mehta, Fridtjof Lund-Johansen, Bernard Fox, Walter Urba, Rachel E. Sanborn, Traci Hilton, Frank Bedu-Addo, Greg Conn, Michael King, Panna Dutta, Robert Shepard, Mark Einstein, Sylvia Adams, Ena Wang, Ping Jin, Yelena Novik, Debra Morrison, Ruth Oratz, Franco M. Marincola, David Stroncek, Judith Goldberg, Sandra Demaria, Silvia C. Formenti, Jérôme Galon, Bernhard Mlecnik, Florence Marliot, Fang-Shu Ou, Carlo B. Bifulco, Alessandro Lugli, Inti Zlobec, Tilman T. Rau, Iris D. Nagtegaal, Elisa Vink-Borger, Arndt Hartmann, Carol Geppert, Michael H. Roehrl, Prashant Bavi, Pamela S. Ohashi, Julia Y. Wang, Linh T. Nguyen, SeongJun Han, Heather L. MacGregor, Sara Hafezi-Bakhtiari, Bradley G. Wouters, Yutaka Kawakami, Boryana Papivanova, Mingli Xu, Tomonobu Fujita, Shoichi Hazama, Nobuaki Suzuki, Hiroaki Nagano, Kiyotaka Okuno, Kyogo Itoh, Eva Zavadova, Michal Vocka, Jan Spacek, Lubos Petruzelka, Bohuslav Konopasek, Pavel Dundr, Helena Skalova, Toshihiko Torigoe, Noriyuki Sato, Tomohisa Furuhata, Ichiro Takemasa, Marc Van den Eynde, Anne Jouret-Mourin, Jean-Pascal Machiels, Tessa Fredriksen, Lucie Lafontaine, Bénédicte Buttard, Sarah Church, Pauline Maby, Helen Angell, Mihaela Angelova, Angela Vasaturo, Gabriela Bindea, Anne Berger, Christine Lagorce, Prabhu S. Patel, Hemangini H. Vora, Birva Shah, Jayendrakumar B. Patel, Kruti N. Rajvik, Shashank J. Pandya, Shilin N. Shukla, Yili Wang, Guanjun Zhang, Giuseppe V. Masucci, Emilia K. Andersson, Fabio Grizzi, Luigi Laghi, Gerardo Botti, Fabiana Tatangelo, Paolo Delrio, Gennaro Cilberto, Paolo A. Ascierto, Franco Marincola, Daniel J. Sargent, Bernard A. Fox, Alain Algazi, Katy Tsai, Michael Rosenblum, Prachi Nandoskar, Robert H. I. Andtbacka, Amy Li, John Nonomura, Kathryn Takamura, Mary Dwyer, Erica Browning, Reneta Talia, Chris Twitty, Sharron Gargosky, Jean Campbell, Carmen Ballesteros-Merino, Carlo B. Bifulco, Bernard Fox, Mai Le, Robert H. Pierce, Adil Daud, Robyn Gartrell, Douglas Marks, Edward Stack, Yan Lu, Daisuke Izaki, Kristen Beck, Dan Tong Jia, Paul Armenta, Ashley White-Stern, Yichun Fu, Zoe Blake, Howard L. Kaufman, Bret Taback, Basil Horst, Yvonne M. Saenger, Steven Leonardo, Keith Gorden, Ross B. Fulton, Kathryn Fraser, Takashi O. Kangas, Richard Walsh, Kathleen Ertelt, Jeremy Graff, Mark Uhlik, Jennifer S. Sims, Liang Lei, Takashi Tsujiuchi, Jeffrey N. Bruce, Peter Canoll, Anthony W Tolcher, Evan W Alley, Gurunadh Chichili, Jan E Canoll, Paul Moore, Ezio Bonvini, Syd Johnson, Sadhna Shankar, James Vasselli, Jon Wigginton, John Powderly

**Affiliations:** 1 Definiens AG, Munich, Bayern Germany; 2 MedImmune, Gaithersburg, MD USA; 3 Dana-Farber Cancer Institute, Boston, MA USA; 4 Erasmus MC Cancer Institute, Rotterdam, Netherlands; 5 Abramson Cancer Center of the University of Pennsylvania, Philadelphia, PA USA; 6 CHU de Québec-Université Laval, Québec, QC Canada; 7 Asan Medical Center and University of Ulsan College of Medicine, Seoul, South Korea; 8 University of California, San Francisco, San Francisco, CA USA; 9 Comprehensive Cancer Centers of Nevada, Las Vegas, NV USA; 10 Fundación Instituto Valenciano de Oncología, Valencia, Spain; 11 Smilow Cancer Hospital at Yale University, New Haven, CT USA; 12 Fondazione IRCCS Istituto Nazionale dei Tumori, Milan, Italy; 13 Radboud University Medical Center, Nijmegen, Netherlands; 14 Westmead Hospital and Macquarie University, Sydney, NSW Australia; 15 Univeristy of Southern California Norris Comprehensive Cancer Center and Hospital, Los Angeles, CA USA; 16 Hôpital Saint-Louis, Paris, France; 17 San Camillo Forlanini Hospital, Rome, Italy; 18 Merck & Co., Inc, Kenilworth, NJ USA; 19 Memorial Sloan Kettering Cancer Center, New York, NY USA; 20 University of Texas MD Anderson Cancer Center, Houston, TX USA; 21 Memorial Sloan Kettering Cancer Center, New York, NY USA; 22 START Madrid, Centro Integral Oncológico Clara Campal, Madrid, Spain; 23 Yale Cancer Center, New Haven, CT USA; 24 Istituto Nazionale dei Tumori-Università degli Studi di Milano, Milano, Lombardia Italy; 25 Dana-Farber Cancer Institute, Boston, MA USA; 26 Comprehensive Cancer Center, Helsinki University Hospital and University of Helsinki, Helsinki, Finland; 27 Emory Winship Cancer Institute, Atlanta, GA USA; 28 Duke University Medical Center, Durham, NC USA; 29 Sidney Kimmel Comprehensive Cancer Center, Johns Hopkins University, Baltimore, MD USA; 30 Oregon Health & Science University, Portland, OR USA; 31 Beatson West of Scotland Cancer Centre, Glasgow, UK; 32 Department of Medical Oncology, Tennessee Oncology, Sarah Cannon Research Institute, Nashville, TN USA; 33 Heidelberg University Hospital, Heidelberg, Baden-Wurttemberg Germany; 34 Vanderbilt-Ingram University Medical Center, Nashville, TN USA; 35 H. Lee Moffitt Cancer Center, Tampa, FL USA; 36 Istituto Nazionale Tumori Fondazione Pascale, Naples, Italy; 37 Bristol-Myers Squibb, Princeton, NJ USA; 38 Compugen Ltd, Holon, Israel; 39 Compugen Inc, USA, South San Francisco, CA USA; 40 Johns Hopkins University, Baltimore, MD USA; 41 Bloomberg ~ Kimmel Institute for Cancer Immunotherapy, Johns Hopkins University, Baltimore, MD USA; 42 Earle A. Chiles Research Institute, Robert W. Franz Cancer Center, Providence Portland Medical Center, Portland, OR USA; 43 Johns Hopkins Sidney Kimmel Comprehensive Cancer Center, Baltimore, MD USA; 44 Dana-Farber Cancer Institute, Boston, MA USA; 45 Memorial Sloan Kettering Cancer Center, New York, NY USA; 46 Massachusetts General Hospital, Boston, MA USA; 47 University of Pittsburg, Pittsburgh, PA USA; 48 Earle A. Chiles Research Institute, Providence Cancer Center, Portland, OR USA; 49 University of Chicago Medical Center, Chicago, IL USA; 50 The Sidney Kimmel Comprehensive Cancer Center, Johns Hopkins University School of Medicine, Lutherville, MD USA; 51 Bristol-Myers Squibb, Princeton, NJ USA; 52 Augusta University, Augusta, GA USA; 53 University of Texas MD Anderson Cancer Center, Houston, TX USA; 54 Cell Design Labs, San Francisco, CA USA; 55 Amgen, San Francisco, CA USA; 56 University of California San Francisco, San Francisco, CA USA; 57 Providence Portland Medical Center, Portland, OR USA; 58 UbiVac, Portland, OR USA; 59 Earle A. Chiles Research Institute, Robert W. Franz Cancer Center, Providence Portland Medical Center, Portland, OR, Portland, OR USA; 60 Earle A. Chiles Research Institute, Providence Cancer Center, Portland, OR USA; 61 ImaginAb Inc, Inglewood, CA USA; 62 AdicetBio, Inc, Menlo Park, CA USA; 63 Medical Oncology Department, Massachusetts General Hospital, Boston, MA USA; 64 Hess Center for Science and Medicine, Tisch Cancer Institute, New York, NY USA; 65 Simon Cancer Center, Indiana University, Indianapolis, IN USA; 66 H. Lee Moffitt Cancer Center, Tampa, FL USA; 67 Department of Biostatistics & Computational Biology, Boston, MA USA; 68 Department of Medical Oncology, City Of Hope, Duarte, CA USA; 69 Biodesix, Inc, Boulder, CO USA; 70 Beth Israel Deaconess Medical Center, Boston, MA USA; 71 Case Western Reserve University, Cleveland, OH USA; 72 The Ohio State University, Columbus, OH USA; 73 Texas Oncology-Baylor Charles A. Sammons Cancer Center, Dallas, TX USA; 74 Earle A. Chiles Research Institute, Providence Cancer Center, Portland, OR USA; 75 Massachusetts General Hospital Cancer Center, Boston, MA USA; 76 Roswell Park Cancer Institute, Buffalo, NY USA; 77 Wake Forest Baptist Medical Center, Winston Salem, NC USA; 78 Levine Cancer Institute, Carolinas HealthCare System, Charlotte, NC USA; 79 Loyola University Medical Center, Maywood, IL USA; 80 Georgetown-Lombardi Comprehensive Cancer Center, Washington, DC USA; 81 Rutgers Cancer Institute of New Jersey, New Brunswick, NJ USA; 82 Robert Lurie Comprehensive Cancer Center of Northwestern University, Chicago, IL USA; 83 Amgen Inc, Thousand Oaks, CA USA; 84 Roswell Park Cancer Institute, Buffalo, NY USA; 85 University of Iowa Hospitals and Clinics, Iowa City, IA USA; 86 University of Utah, Huntsman Cancer Institute, Salt Lake City, UT USA; 87 California Pacific Melanoma Center, San Francisco, CA USA; 88 The Angeles Clinic & Research Institute, Los Angeles, CA USA; 89 Rutgers Cancer Institute of New Jersey, New Brunswick, NJ USA; 90 Veana Therapeutics Inc, Portland, OR USA; 91 Earle A. Chiles Research Institute, Providence Cancer Center, Portland, OR USA; 92 Earle A. Chiles Research Institute, Robert W. Franz Cancer Center, Providence Portland Medical Center, Portland, OR USA; 93 Icahn School of Medicine at Mount Sinai, New York, NY USA; 94 Celldex Therapeutics, Hampton, NJ USA; 95 Oncovir, Inc, Washington, DC USA; 96 Mount Sinai School of Medicine, New York, NY USA; 97 University of California, Davis, Sacramento, CA USA; 98 University of California Davis Comprehensive Cancer Center, Sacramento, CA USA; 99 The Ohio State University Wexner Medical Center and The OSU James Comprehensive Cancer Center, Columbus, OH USA; 100 The Ohio State University, Columbus, OH USA; 101 Yale University, New Haven, CT USA; 102 The Pennsylvania State University College of Medicine, Hershey, PA USA; 103 Roswell Park Cancer Institute, Buffalo, NY USA; 104 Earle A. Chiles Research Institute, Portland, OR USA; 105 Providence Cancer Center, Portland, OR USA; 106 Medical University of South Carolina, Charleston, SC USA; 107 XOMA Corporation, Berkeley, CA USA; 108 Medical University of South Carolina, Charleston, SC USA; 109 Altor BioScience Corporation, Miramar, FL USA; 110 Georgia Cancer Center, Augusta, GA USA; 111 Qiagen, Frederick, MD USA; 112 Sanford Cancer Center, Sioux Falls, SD USA; 113 Sanford Roger Maris Cancer Center, Fargo, ND USA; 114 Sanford Health, Sioux Falls, SD USA; 115 Moores Cancer Center, University of California, San Diego, La Jolla, CA USA; 116 NantKwest, Inc, Culver City, CA USA; 117 Weill Cornell Medicine, New York, NY USA; 118 Memorial Sloan Kettering Cancer Center, New York, NY USA; 119 New York University School of Medicine, New York, NY USA; 120 Department of Radiation Oncology, Weill Cornell Medicine, New York, NY USA; 121 Laboratory of Tumor Immunology and Biology, Center for Cancer Research, National Cancer Institute, Bethesda, MD USA; 122 Fischell Department of Bioengineering, University of Maryland - College Park, College Park, MD USA; 123 UbiVac, Portland, OR USA; 124 Section of Hematology/Oncology, Louisiana State University, New Orleans, LA USA; 125 Robert W. Franz Cancer Research Center, Earle A. Chiles Research Institute, Providence Cancer Center, Portland, OR USA; 126 Louisiana State University Stanley S. Scott Cancer Center, New Orleans, LA USA; 127 PPMC, Portland, OR USA; 128 Providence Cancer Center, Portland, OR USA; 129 Providence Medical Center, Portland, OR USA; 130 Earle A. Chiles Research Institute, Providence Cancer Center, Portland, OR USA; 131 Providence Medical Center, Portland, OR USA; 132 UbiVac, Providence Medical Center, Portland, OR USA; 133 Oslo University Hospital, Oslo, Norway; 134 PDS Biotech, New Brunswick, NJ USA; 135 PDS Biotech, Miami Beach, FL USA; 136 Rutgers, NJ Medical School, Newark, NJ USA; 137 Perlmutter Cancer Center, New York University School of Medicine, NYC, NY USA; 138 Sidra Medical and Research Center, Doha, Qatar; 139 National Institutes of Health Clinical Center Department of Transfusion Medicine, Bethesda, MD USA; 140 National Institutes of Health Clinical Center, Bethesda, MD USA; 141 Weill Cornell Medicine, Department of Radiation Oncology, New York, NY USA; 142 INSERM, Université Pierre et Marie Curie, Université Paris Descartes, Paris, France; 143 Mayo Clinic, Rochester, MN USA; 144 Earle A. Chiles Research Institute, Providence Cancer Center, Portland, Oregon USA; 145 Institute of Pathology, University of Bern, Bern, Switzerland; 146 Radboud University Nijmegen Medical Center, Nijmegen, Netherlands; 147 University Erlangen-Nürnberg, Erlangen, Germany; 148 Princess Margaret Cancer Centre, University Health Network, Toronto, ON Canada; 149 Division of Cellular Signaling, Institute for Advanced Medical Research, Keio University School of Medicine, Tokyo, Japan; 150 Department of Gastroenterological, Breast and Endocrine Surgery, Yamaguchi University Graduate School of Medicine, Ube, Japan; 151 Department of Surgery, Kinki University Faculty of Medicine, Osaka-Sayama, Japan; 152 Division of Clinical Research, Research Center for Innovative Cancer Therapy, Kurume University School of Medicine, Kurume, Japan; 153 First Faculty of Medicine, Charles University and General University Hospital, Prague, Czech Republic; 154 Sapporo Medical University, Sapporo, Japan; 155 Institut Roi Albert II, Cliniques universitaires St-Luc, Université Catholique de Louvain, Brussels, Belgium; 156 The Gujarat Cancer & Research Institute, Ahmedabad, India; 157 Institute for Cancer Research, Xi’an Jiaotong University, Xi’an, China; 158 Karolinska Institutet, Karolinska University, Stockholm, Sweden; 159 Humanitas Clinical and Research Center, Rozzano, Italy; 160 Istituto Nazionale Tumori Fondazione Pascale, Naples, Italy; 161 Sidra Medical and Research Center, Doha, Qatar; 162 University of California, San Francisco, San Francisco, CA USA; 163 Huntsman Cancer Institute, University of Utah, Salt Lake City, UT USA; 164 OncoSec Medical Inc., San Diego, CA USA; 165 Robert W. Franz Cancer Research Center, Earle A. Chiles Research Institute, Providence Cancer Center, Portland, Oregon, USA; 166 Doctor Hope, LLC, San Diego, CA USA; 167 Columbia University Medical Center, New York, NY USA; 168 Perkin Elmer, Hopkinton, MA USA; 169 Columbia University, New York, NY USA; 170 Columbia University College of Physicians and Surgeons, New York, NY USA; 171 Rutgers Cancer Institute of New Jersey, New Brunswick, NJ USA; 172 New York Presbyterian/Columbia University Medical Center, New York, NY USA; 173 Biothera Pharmaceuticals Inc., Eagan, MN USA; 174 Columbia University Medical Center, New York, NY USA; 175 START - South Texas Accelerated Research Therapeutics, LLC, San Antonio, TX USA; 176 Penn Presbyterian Medical Center, University of Pennsylvania, Philadelphia, PA USA; 177 MacroGenics, Inc., Rockville, MD USA; 178 MacroGenics, Inc., South San Francisco, CA USA; 179 Carolina BioOncology Institute, PLLC, Huntersville, NC USA

## Biomarkers and Immune Monitoring

### O1 Combinatorial CD8+ and PD-L1+ cell densities correlate with response and improved survival in non-small cell lung cancer (NSCLC) patients treated with durvalumab

#### Sonja Althammer^1^, Keith Steele^2^, Marlon Rebelatto^2^, Tze Heng Tan^1^, Tobias Wiestler^1^, Guenter Schmidt^1^, Brandon Higgs^2^, Xia Li^2^, Li Shi^2^, Xiaoping Jin^2^, Joyce Antal^2^, Ashok Gupta^2^, Koustubh Ranade^2^, Gerd Binning^1^

##### ^1^Definiens AG, Munich, Bayern, Germany; ^2^MedImmune, Gaithersburg, MD, USA

###### **Correspondence:** Brandon Higgs (higgsb@medimmune.com)

**Background**

Immunotherapies have improved patient responses and survival, though not all patients benefit. Effective biomarkers may help to improve outcomes. Durvalumab is a human IgG1 monoclonal antibody that inhibits PD-L1 binding to PD-1 and CD80, restoring antitumor immunity [1, 2]. PD-L1 expression on tumor or tumor-infiltrating immune cells measured manually with different immunohistochemistry (IHC) assays can enrich for patients responding to anti-PD-1/PD-L1 agents. Tumor-infiltrating cytotoxic CD8+ T cells may also have potential predictive utility for therapeutic response. We explored automated image analysis and pattern recognition of tumor biopsies to determine whether CD8+ and PD-L1+ cell densities could better identify patients most likely to respond to durvalumab than PD-L1 IHC alone.

**Methods**

CP1108/NCT01693562 was a nonrandomized phase I/II trial evaluating durvalumab in advanced NSCLC and other solid tumors [3]. By 29APR2016, 304 previously treated NSCLC patients, median 3 prior lines, received 10 mg/kg of durvalumab q2w ≤12 months. Baseline archived or fresh tumor biopsies were analyzed for PD-L1 (Ventana/SP263) and CD8 (Ventana/SP239) by IHC. For the marker combination, slides were scored using the product of PD-L1+ and CD8+ cell densities with Definiens’ Developer XD 2.1.4 software. For PD-L1 alone, ≥25% tumor cells stained for PD-L1 at any intensity were scored positive. Clinical outcomes (ORR, PFS and OS) were analysed based on CD8+ and PD-L1+ densities (n = 163 available) and PD-L1 alone in pre-treatment biopsies using a discovery (n = 84) and validation (n = 79) set. Datasets were matched on baseline PD-L1 status, histology, ECOG, lines of therapy, and response.

**Results**

**Fig. 1 (abstract O1).**
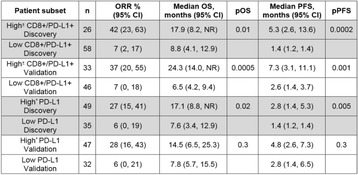
Clinical outcomes in CD8+/PD-L1+ or PD-L1 NSCLC patient subsets

Patients with high pretreatment CD8+ and PD-L1+ densities (prevalence = 36%) had better ORR, OS, and PFS compared to those with low CD8+ and PD-L1+ densities (Fig. 1), as well as high PD-L1 expression alone.

**Conclusions**

Automated image analysis of CD8+ and PD-L1+ cell densities in baseline tumor biopsies may identify patients with improved outcomes to durvalumab.

**Trial Registration**

ClinicalTrials.gov identifier NCT01693562.

**References**

1. MedImmune/AstraZeneca. Data on file.

2. Ibrahim R, Stewart R, Shalabi A: **PD-L1 blockade for cancer treatment: MEDI4736.** *Semin Oncol* 2015*,* **42**:474–483.

3. Rizvi NA, Brahmer JR, Ou SHI, Segal NH, Khleif S, Hwu WJ, *et al:* **Safety and clinical activity of MEDI4736, an anti-programmed cell death-ligand 1 (PD-L1 antibody, in patients with non-small lung cancer (NSCLC).** *J Clin Oncol* 2015*,* **33**(Suppl.):Abstract 8032.

## Clinical Trials: Cutting-Edge (Completed Trials)

### O2 Keynote-045: open-label, phase III study of pembrolizumab versus investigator’s choice of paclitaxel, docetaxel, or vinflunine for previously treated advanced urothelial cancer

#### Joaquim Bellmunt,^1^ Ronald de Wit,^2^ David J Vaughn,^3^ Yves Fradet,^4^ Jae Lyun Lee,^5^ Lawrence Fong,^6^ Nicholas J Vogelzang,^7^ Miguel A Climent,^8^ Daniel P Petrylak,^9^ Toni K Choueiri,^1^ Andrea Necchi,^10^ Winald Gerritsen,^11^ Howard Gurney,^12^ David I Quinn,^13^ Stéphane Culine,^14^ Cora N Sternberg,^15^ Yabing Mai,^16^ Markus Puhlmann,^16^ Rodolfo F Perini,^16^ Dean F Bajorin^17^

##### ^1^Dana-Farber Cancer Institute, Boston, MA, USA; ^2^Erasmus MC Cancer Institute, Rotterdam, Netherlands; ^3^Abramson Cancer Center of the University of Pennsylvania, Philadelphia, PA, USA; ^4^CHU de Québec-Université Laval, Québec, QC, Canada; ^5^Asan Medical Center and University of Ulsan College of Medicine, Seoul, South Korea; ^6^University of California, San Francisco, San Francisco, CA, USA; ^7^Comprehensive Cancer Centers of Nevada, Las Vegas, NV, USA; ^8^Fundación Instituto Valenciano de Oncología, Valencia, Spain; ^9^Smilow Cancer Hospital at Yale University, New Haven, CT, USA; ^10^Fondazione IRCCS Istituto Nazionale dei Tumori, Milan, Italy; ^11^Radboud University Medical Center, Nijmegen, Netherlands; ^12^Westmead Hospital and Macquarie University, Sydney, NSW, Australia; ^13^Univeristy of Southern California Norris Comprehensive Cancer Center and Hospital, Los Angeles, CA, USA; ^14^Hôpital Saint-Louis, Paris, France; ^15^San Camillo Forlanini Hospital, Rome, Italy; ^16^Merck & Co., Inc., Kenilworth, NJ, USA; ^17^Memorial Sloan Kettering Cancer Center, New York, NY, USA

**Background**

There is no standard second-line therapy for advanced urothelial cancer. Although paclitaxel, docetaxel, and vinflunine are commonly used, they provide limited clinical benefit. KEYNOTE-045 compared the efficacy and safety of the anti–PD-1 antibody pembrolizumab versus investigator-choice chemotherapy as second-line therapy for advanced urothelial cancer that progressed or recurred following first-line platinum-based chemotherapy.

**Methods**

Eligible patients were enrolled regardless of PD-L1 expression and randomized 1:1 to pembrolizumab 200 mg Q3W for 24 months or investigator’s choice of paclitaxel 175 mg/m^2^ Q3W, docetaxel 75 mg/m^2^ Q3W, or vinflunine 320 mg/m^2^ Q3W. Randomization was stratified by ECOG PS (0/1 vs 2), liver metastases (yes vs no), hemoglobin level (<10 vs ≥10 g/dL), and time from last chemotherapy dose (<3 vs ≥3 months). The study had a group sequential design to control for type I error. Primary endpoints were OS and PFS (RECIST v1.1 by blinded, independent central review). ORR was a key secondary endpoint. Differences in OS and PFS were assessed in the intention-to-treat population using the stratified log-rank test

**Results**

Between November 5, 2014 and November 13, 2015, 542 patients from 29 countries were enrolled: 270 in the pembrolizumab arm, 272 in the chemotherapy arm. As of September 7, 2016, median follow-up was 9.0 months; 49 (18.4%) patients remained on pembrolizumab and 3 (1.2%) patients remained on chemotherapy. Baseline characteristics were generally balanced between arms, with 87.3% with visceral disease, 34.3% with liver metastases, 1.1% with ECOG PS 2, 81.5% with hemoglobin ≥10 g/dL, and 38.2% with <3 months since most recent chemotherapy. Pembrolizumab significantly improved OS over chemotherapy (HR 0.73, *P* = 0.0022; median 10.3 vs 7.4 months) (Table 1). There was no difference in PFS (HR 0.98, *P* = 0.42) (Table 1). ORR was significantly improved with pembrolizumab (21.1% vs 11.4%) (Table 1). Pembrolizumab was associated with fewer any-grade (60.9% vs 90.2%) grade 3-5 treatment-related AEs (15.0% vs 49.4%). 4 patients in each arm died due to treatment-related AEs.

**Conclusions**

Pembrolizumab demonstrated a statistically significant OS benefit over chemotherapy in the second-line advanced urothelial cancer setting, making it the first therapy to demonstrate a survival benefit over an active comparator in this population. The superior OS combined with the lower rate of any-grade and high-grade treatment-related AEs support pembrolizumab as a new standard of care for advanced urothelial cancer that progressed on/after platinum-based chemotherapy.

**Trial registration**

ClinicalTrials.gov identifier: NCT02256436

**Table 1 (abstract O2):** Efficacy in KEYNOTE-045

End point	Pembrolizumab N = 270	Chemotherapy N = 272
OS, no. of events	155	179
Median (95 % CI), months	10.3 (8.0-11.8)	7.4 (6.1-8.3)
HR (95 % CI)	0.73 (0.59-0.91); *P* = 0.0022	
PFS, no. of events	218	219
Median (95 % CI), months	2.1 (2.0-2.2)	3.3 (2.3-3.5)
HR (95 % CI)	0.98 (0.81-1.19); *P* = 0.42	
ORR (95 % CI)	21.1 % (16.4-26.5)	11.4 % (7.9-15.8)
Treatment difference, % (95 % CI)	9.6 (3.5-15.9); *P* = 0.0011	

### O3 Efficacy and safety of nivolumab plus ipilimumab in metastatic urothelial carcinoma: first results from the phase I/II CheckMate 032 study

#### Padmanee Sharma^1^, Margaret K Callahan^2^, Emiliano Calvo^3^, Joseph W Kim^4^, Filipo de Braud^5^, Patrick A Ott^6^, Petri Bono^7^, Rathi N Pillai^8^, Michael Morse^9^, Dung T Le^10^, Matthew Taylor^11^, Pavlina Spilliopoulou^12^, Johanna Bendell^13^, Dirk Jaeger^14^, Emily Chan^15^, Scott J Antonia^16^, Paolo A Ascierto^17^, Delphine Hennicken^18^, Marina Tschaika^18^, Alex Azrilevich^18^, Jonathan Rosenberg^2^

##### ^1^University of Texas MD Anderson Cancer Center, Houston, TX, USA; ^2^Memorial Sloan Kettering Cancer Center, New York, NY, USA; ^3^START Madrid, Centro Integral Oncológico Clara Campal, Madrid, Spain; ^4^Yale Cancer Center, New Haven, CT, USA; ^5^Istituto Nazionale dei Tumori-Università degli Studi di Milano, Milano, Lombardia, Italy; ^6^Dana-Farber Cancer Institute, Boston, MA, USA; ^7^Comprehensive Cancer Center, Helsinki University Hospital and University of Helsinki, Helsinki, Finland; ^8^Emory Winship Cancer Institute, Atlanta, GA, USA; ^9^Duke University Medical Center, Durham, NC, USA; ^10^Sidney Kimmel Comprehensive Cancer Center at Johns Hopkins University, Baltimore, MD, USA; ^11^Oregon Health & Science University, Portland, OR, USA; ^12^Beatson West of Scotland Cancer Centre, Glasgow, United Kingdom; ^13^Sarah Cannon Research Institute and Department of Medical Oncology, Tennessee Oncology, Nashville, TN, USA; ^14^Heidelberg University Hospital, Heidelberg, Baden-Wurttemberg, Germany; ^15^Vanderbilt-Ingram University Medical Center, Nashville, TN, USA; ^16^H. Lee Moffitt Cancer Center, Tampa, FL, USA; ^17^Istituto Nazionale Tumori Fondazione Pascale, Naples, Italy, Napoli, Italy; ^18^Bristol-Myers Squibb, Princeton, NJ, USA

###### **Correspondence:** Padmanee Sharma (padsharma@mdanderson.org)

**Background**

Nivolumab is a programmed death-1 (PD-1) immune checkpoint inhibitor associated with clinical benefit in previously treated patients with metastatic urothelial carcinoma [1]. Preclinical and clinical data indicate that the combination of nivolumab plus ipilimumab, an anti-cytotoxic T-lymphocyte antigen-4 (CTLA-4) antibody, can improve antitumor activity in other tumor types. Here, we report the first efficacy and safety results of combined nivolumab plus ipilimumab given at two different dosing schedules in CheckMate 032 , an open-label, multicenter, phase I/II study of patients with metastatic urothelial carcinoma who progressed after prior platinum-based therapy.

**Methods**

Patients with locally advanced or metastatic urothelial carcinoma previously treated with platinum-based therapy were included in the study. Patients were treated with either of two combination schedules, nivolumab 1 mg/kg + ipilimumab 3 mg/kg (N1I3) or nivolumab 3 mg/kg + ipilimumab 1 mg/kg (N3I1) every 3 weeks for four cycles, followed by nivolumab 3 mg/kg every 2 weeks; or they were treated with nivolumab monotherapy 3 mg/kg (N3) every 2 weeks. All patients were treated until disease progression or unacceptable toxicity. The primary endpoint was investigator-assessed objective response rate (ORR) by RECIST v1.1. Secondary endpoints included safety and duration of response (DoR).

**Results**

Minimum follow-up was 3.9 months in the N1I3 (n = 26) group, 14.5 months in the N3I1 group (n = 104), and 13.8 months in N3 group (n = 78). ORR was 38.5% (95% confidence interval [CI], 20.2-59.4), 26.0% (95% CI, 17.9-35.5), and 25.6% (95% CI, 16.4-36.8) in the N1I3, N3I1, and N3 groups, respectively. Median DoR has not been reached in any treatment group. The frequency of drug-related grade 3-4 adverse events was 30.8% (N1I3), 31.7% (N3I1), and 23.1% (N3). Treatment-related adverse events led to discontinuation in 7.7% (N1I3), 13.5% (N3I1), and 3.8% (N3) of patients. One death was reported in the N3I1 group (pneumonitis) and two deaths were reported in the N3 group (pneumonitis and thrombocytopenia).

**Conclusions**

Second-line treatment with N1I3 may provide the most favorable benefit-risk ratio among the regimens studied. If these interim results are confirmed with longer follow-up, further development of the N1I3 combination in metastatic urothelial carcinoma is warranted.

**Trial Registration**

ClinicalTrials.gov identifier NCT01928394.

**References**

1. Sharma P, Bono P, Kim JW, *et al:* **Efficacy and safety of nivolumab monotherapy in metastatic urothelial cancer (mUC): Results from the phase I/II CheckMate 032 study.** *J Clin Oncol* 2016, **34**(15 suppl): Abstract 4501.

## Coinhibition & Costimulation

### O4 Computational identification, functional characterization, and antibody blockade of a new immune checkpoint in the TIGIT family of interacting molecules

#### Ofer Levy^1^, Christopher Chan^2^, Gady Cojocaru^1^, Spencer Liang^2^, Eran Ophir^1^, Sudipto Ganguly^3^, Amir Toporik^1^, Maya Kotturi^2^, Tal Fridman Kfir^1^, Benjamin M. Murter^3^, Kathryn Logronio^2^, Liat Dassa^1^, Ling Leung^2^, Shirley Greenwald^1^, Meir Azulay^1^, Sandeep Kumar^2^, Zoya Alteber^1^, Xiaoyu Pan^4^, Arthur Machlenkin^1^, Yair Benita^1^, Andrew W. Drake^2^, Ayelet Chajut^1^, Ran Salomon^1^, Ilan Vankin^1^, Einav Safyon^1^, John Hunter^2^, Zurit Levine^1^, Mark White^2^

##### ^1^Compugen Ltd., Holon, Israel; ^2^Compugen Inc, USA, South San Francisco, CA, USA; ^3^Johns Hopkins University, Baltimore, MD, USA; ^4^Bloomberg ~ Kimmel Institute for Cancer Immunotherapy, Johns Hopkins University, Baltimore, MD, USA

###### **Correspndence:** John Hunter (johnh@cgen.com)

**Background**

While antibody blockade of the CTLA-4 and PD-1 pathways has emerged as an effective treatment modality for cancer, the majority of patients do not derive long-term benefit, suggesting a need for targeting of additional immune checkpoints. Employing our unique computational algorithms to define new members of the B7/CD28 family, we identified PVRIG, which is expressed by multiple subsets of T and NK cells. We report here its expression pattern, functional characterization, and anti-tumor activity of blocking antibodies targeting this molecule.

**Methods**

Utilizing Compugen’s Predictive Discovery platform we identified PVRIG as a potential novel immune checkpoint, after which a retroviral cell screening library was used to identify its cognate binding counterpart. Target effects on T cell modulation were assessed with primary and tumor-derived T cell assays, taking advantage of target overexpression, knockdown, and antagonist antibody approaches. Antibodies against the human protein were screened for their ability to enhance T cell activation *in vitro*, while antibodies targeting the mouse orthologue were assessed *in vivo* for effects on tumor growth inhibition in syngeneic models.

**Results**

A PVRIG-Fc-fusion protein was found to bind PVRL2, with binding specificity confirmed both by ELISA and flow cytometry analysis. PVRIG demonstrated unique expression kinetics upon T cell activation, with detection of the target on memory T cells, as well as on NK cells and γδ T cells. A panel of high affinity human antibodies with the ability to block interaction of PVRIG with PVRL2 were generated, which when tested *in vitro* were shown to enhance activation of both primary CD4+ and tumor-derived CD8+ T cells through a PVRL2-dependent mechanism. The lead antibody, COM-701, is currently in preclinical development. Since COM-701 is not mouse cross-reactive, *in vivo* studies were conducted with a surrogate blocking anti-mouse PVRIG antibody. When combined with anti-PD-L1 blockade, anti-mouse PVRIG inhibits growth of established tumors in both the CT26 and MC38 colorectal cancer models. Combination testing with additional immune checkpoint inhibitors, as well as in PVRIG knockout mice, is ongoing.

**Conclusions**

We describe the identification of PVRIG as a novel immune checkpoint on T cells, as well the development of a high affinity antagonistic antibody, COM-701, that is currently in preclinical development. COM-701 is able to enhance human T cell activation, and a surrogate antibody with similar characteristics shows synergy with PD-L1 *in vivo* in multiple syngeneic models. Overall, our data demonstrate the utility of targeting PVRIG in addition to other B7 family checkpoints for the treatment of cancer.

## Combinations: Immunotherapy/Immunotherapy

### O5 Preliminary efficacy from a phase I/II study of the natural killer cell–targeted antibody lirilumab in combination with nivolumab in squamous cell carcinoma of the head and neck

#### Rom Leidner^1^, Hyunseok Kang^2^, Robert Haddad^3^, Neil H Segal^4^, Lori J Wirth^5^, Robert L Ferris^6^, F Stephen Hodi^3^, Rachel E Sanborn^7^, Thomas F Gajewski^8^, William Sharfman^9^, Dan McDonald^10^, Shivani Srivastava^10^, Xuemin Gu^10^, Penny Phillips^10^, Chaitali Passey^10^, Tanguy Seiwert^8^

##### ^1^Earle A. Chiles Research Institute, Robert W. Franz Cancer Center, Providence Portland Medical Center, Portland, OR, USA; ^2^Johns Hopkins Sidney Kimmel Comprehensive Cancer Center, Baltimore, MD, USA; ^3^Dana-Farber Cancer Institute, Boston, MA, USA; ^4^Memorial Sloan Kettering Cancer Center, New York, NY, USA; ^5^Massachusetts General Hospital, Boston, MA, USA; ^6^University of Pittsburg, Pittsburgh, PA, USA; ^7^Earle A. Chiles Research Institute, Providence Cancer Center, Portland, OR, USA; ^8^University of Chicago Medical Center, Chicago, IL, USA; ^9^The Sidney Kimmel Comprehensive Cancer Center, Johns Hopkins University School of Medicine, Lutherville, MD, USA; ^10^Bristol-Myers Squibb, Princeton, NJ, USA

###### **Correspondence:** Rom Leidner (rom.leidner@providence.org)

**Background**

Natural killer (NK) cells and the innate immune system play a critical role in immunosurveillance, control of tumor growth, and metastasis. NK-cell activation is negatively regulated by inhibitory killer-cell immunoglobulin-like receptors (KIRs); therefore, blocking KIR function may potentiate an anti-tumor immune response and complement other immuno-oncology therapies that enhance T cell activity. We present preliminary efficacy results in patients with squamous cell carcinoma of the head and neck (SCCHN) from a phase I/II study of lirilumab, a fully human monoclonal antibody that blocks inhibitory KIRs on NK cells, in combination with nivolumab, a fully human IgG4 monoclonal antibody that targets the PD-1 receptor, in patients with solid tumors (NCT01714739).

**Methods**

During dose escalation, patients with advanced solid tumors who progressed after ≥ 1 prior therapy received lirilumab 0.1–3.0 mg/kg once every 4 weeks (Q4W) plus nivolumab 3.0 mg/kg Q2W. Cohort expansion was initiated at the maximum dose of lirilumab 3.0 mg/kg Q4W plus nivolumab 3.0 mg/kg Q2W in patients with advanced solid tumors. Key study endpoints include safety (primary), objective response rate (ORR), disease control rate (DCR), duration of response (DOR), and biomarker assessments.

**Results**

**Fig. 2 (abstract O5).**
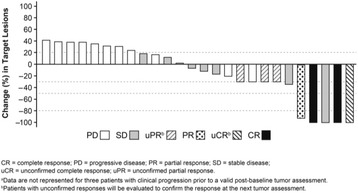
Maximum percent reduction in target lesions from baseline^a^

**Fig. 3 (abstract O5).**
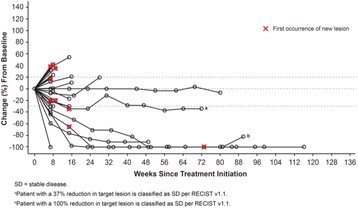
Percent change from baseline in target lesions over time

As of the August 30, 2016 data cutoff, 159 patients were treated with the lirilumab plus nivolumab combination. Treatment-related adverse events (TRAEs) and grade 3–4 TRAEs were reported in 72% (114/159) and 15% (24/159) of patients, respectively. Discontinuations due to TRAEs occurred in 8% (12/159). Of the 41 patients with SCCHN treated, 29 were evaluable for response. In this heavily pretreated, checkpoint inhibitor–naïve group, ORR was 24% (7/29; confirmed and unconfirmed) and DCR was 52% (15/29). Maximum reduction in target lesions are presented in Fig. 2 for 26 patients with available tumor assessments. Two patients classified as stable disease per RECIST v1.1 showed unconventional responses, with 100% and 37% reductions in target lesions. Among evaluable patients, five (17%) had reductions in tumor burden > 80%. Responses appear durable, with the median DOR not reached (Fig. 3). Updated efficacy and preliminary biomarker analyses (including PD-L1 and HPV status) will be presented.

**Conclusions**

Preliminary efficacy of lirilumab plus nivolumab in patients with advanced platinum-refractory SCCHN demonstrates clinical benefit, with encouraging response rates that were deep and durable responses in some patients. This combination demonstrated a manageable safety profile similar to that observed with nivolumab monotherapy. Further evaluation of this novel combination of an NK-cell inhibitor and an immune checkpoint inhibitor is ongoing.

**Trial Registration**

ClinicalTrials.gov identifier: NCT01714739

## Tumor Microenvironment

### 06 Phase II study of intratumoral plasmid interleukin 12 (pIL-12) with electroporation in combination with pembrolizumab in stage III/IV melanoma patients with low tumor infiltrating lymphocytes

#### Alain Algazi^1^, Katy Tsai^1^, Michael Rosenblum^1^, Prachi Nandoskar^1^, Robert HI Andtbacka^2^, Amy Li^1^, John Nonomura^3^, Kathryn Takamura^3^, Mary Dwyer^3^, Erica Browning^3^, Reneta Talia^3^, Chris Twitty^3^, Sharron Gargosky^3^, Jean Campbell^3^, Carmen Ballesteros-Merino^4^, Carlo B. Bifulco^4^, Bernard Fox^4^, Mai Le^5^, Robert H Pierce^3^, Adil Daud^1^

##### ^1^University of California, San Francisco, San Francisco, CA, USA; ^2^Huntsman Cancer Institute, University of Utah, Salt Lake City, UT, USA; ^3^OncoSec Medical Inc., San Diego, CA, USA; ^4^Robert W. Franz Cancer Research Center, Earle A. Chiles Research Institute, Providence Cancer Center, Portland, Oregon, USA; ^5^Doctor Hope, LLC, San Diego, CA, USA

###### **Correspondence:** Sharron Gargosky (sgargosky@oncosec.com)

**Background**

Low tumor infiltrating lymphocytes (TIL) are predictive for poor response to immunotherapy with anti-PD-1/PD-L1 agents. We have shown that melanoma patients with a low frequency of PD-1hiCTLA-4 + TIL are unlikely to respond to pembrolizumab (Daud2016). Intratumoral electroporation of pIL-12 (IT-pIL12-EP) leads to an IFN-g signature suggestive of increased TIL as well as regression in both treated and untreated lesions. We hypothesize that combination IT-pIL12-EP and pembrolizumab will improve clinical outcomes in this low-response population. Preliminary results from a multi-center, phase II, open-label trial testing this hypothesis are presented.

**Methods**

Melanoma stage III/IV patients with accessible lesions were consented and enrolled if they had a TIL status of hiCTLA-4+ in the CD45 + CD8 + CD3+ gate by flow cytometry (FC). Patients were treated with pembrolizumab (200 mg every 3 weeks) concurrently with IT-pIL12-EP on days 1, 5 and 8 every 6 weeks. Patients were evaluated for overall response rate (ORR) every 12 weeks by RECISTv1.1. Pre and post-treatment blood and tumor specimens were collected, and analyzed for immune phenotyping, gene expression, TCR diversity, and changes in the tumor microenvironment with multispectral immunohistochemistry.

**Results**

Interim ORR data is available on 15 patients. 13/15 patients had a frequency of PD-1hi CTLA-4+ TIL of < 22% (low TIL status), phenotypes associated with a low probability of response to anti-PD-1 (Daud 2016). These 15 patients age 39-89 years, were 53% male, 66% stage III and 34% stage IV. Treatment was well tolerated; 38% of adverse events (AE) were classified as treatment site reactions (grade 1-2) that resolved. One SAE of cellulitis resolved with 5d antibiotics. One grade 3 AE of diarrhea resolved with corticosteroids. The ORR was 40% (4CR, 2PR) by RECISTv1.1. Analysis of tumor biopsies and blood demonstrated meaningful immunological changes including an increased number and ratio of CD8+:PD-L1+ and CD8+:FoxP3 + TIL, tumoral RNA signatures indicating an increase in CD8 and IFN-γ-related gene expression and concordant immune phenotypes in the periphery.

**Conclusions**

The combination IT-pIL12-EP with pembrolizumab in patients with an anti-PD-1 non-responsive phenotype engendered a 40% clinical response with associated positive immune-based biomarker data and an excellent safety profile. These data suggests that IT-pIL12-EP modulates the tumor microenvironment to enable an effective anti-PD-1 mAb response in patients otherwise unlikely to respond.

**Acknowledgements**

We thank Merck and Oncosec for supporting this trial with pembrolizumab and IT-pIL-12, respectfully.

**Trial Registration**

ClinicalTrials.gov identifier: NCT02493361

**References**

1. Daud AI, Loo K, Pauli ML, Sanchez-Rodriguez R, Sandoval PM, Taravati K, et al: **Tumor immune profiling predicts response to anti–PD-1 therapy in human melanoma**. *J Clin Invest* 2016, **126(9)**:3447–3452.

## Adoptive Cellular Therapy

### P1 Chemo-immunotherapy with cyclophosphamide and tumor reactive CD4+ T cells lead to destruction of tumor vasculature and eventual tumor eradication

#### Tsadik Habtetsion, Gang Zhou

##### Augusta University, Augusta, GA, USA

###### **Correspondence:** Tsadik Habtetsion (tsadikg855@yahoo.com)

**Background**

CD4^+^ T cells are critical components of anti-tumor immunity and play a pivotal role in orchestrating anti-tumor immune responses. Mounting evidence from preclinical and clinical studies indicates that CD4^+^ T cells in combination with chemotherapy can control tumor growth and recurrence. CD4^+^ T cells are suggested to mediate tumor rejection through mechanisms that include cytotoxic effects on tumor cells, inhibition of angiogenesis, and reprogramming of the tumor microenvironment.

**Methods**

In this project, we set out to study the cellular and molecular mechanisms underlying the therapeutic effect of chemo-immunotherapy in the form of cyclophosphamide (CTX) and tumor specific CD4^+^ T cells in a murine model of colorectal cancer. Mice were injected subcutaneously with colorectal cancer cells. When the tumor reached 140-160 mm^2^ in area, mice were injected with a low dose of cyclophosphamide followed by adoptive transfer of tumor reactive CD4^+^ T cells.

**Results**

In a murine model of colorectal cancer, we show that the combination therapy of CTX and tumor reactive CD4^+^ T cells resulted in enhanced necrosis of tumor cells *in vivo*, leading to eventual eradication of advanced tumors. By using immunofluorescence staining and blood perfusion imaging, we demonstrated that the combination therapy leads to destruction of the established tumor vasculature and reduced blood supply to tumor tissue. Furthermore, we assessed blood vessel permeability in the tumor tissue and found that the combination therapy increased extravasation of Evans blue dye, suggesting an increase in vascular permeability.

**Conclusions**

In summary, our findings suggest that the combination therapy of CTX + CD4^+^ T cells leads to destruction of the tumor vasculature, resulting in extensive necrosis of tumor tissue and eventual tumor regression. These findings may provide new insights into mechanisms of tumor rejection by CD4^+^ T cells.

### P2 Preclinical development of tumor-infiltrating lymphocyte therapy for pancreatic cancer

#### Donastas Sakellariou-Thompson, Cara Haymaker, Caitlin Creasy, Mark Hurd, Naohiro Uraoka, Jaime Rodriguez Canales, Scott Koptez, Patrick Hwu, Anirban Maitra, Chantale Bernatchez

##### University of Texas MD Anderson Cancer Center, Houston, TX, USA

###### **Correspondence:** Donastas Sakellariou-Thompson (dasakellariou@mdanderson.org)

**Background**

Immunotherapy has become an effective cancer therapy, particularly in the case of checkpoint blockade and adoptive T cell therapy (ACT). ACT exploits the presence of tumor-infiltrating lymphocytes (TIL) by exponentially expanding their numbers *ex vivo* and re-infusing them into the patient in an autologous setting. With the effectiveness of TIL therapy already well established in multiple phase II studies in melanoma, there is a push to translate it to other cancers in dire need of improved therapies. Pancreatic ductal adenocarcinoma (PDAC) is one such cancer for which the current therapy, surgery and chemotherapy, provides an overall 5-year survival rate of only 5%. The presence of TIL is correlated with increased survival in PDAC, which suggests that TIL could effectively control the disease and provides a rationale to test TIL therapy in this setting.

**Methods**

To assess the feasibility, we characterized the immune component of PDAC, explored the ability to grow and expand TIL from tumor fragments, and analyzed the clonality of these expanded TIL.

**Results**

Flow cytometry analysis detected low, CD4-rich T cell infiltration. These TIL were able to be expanded *ex vivo* and the addition of an agonistic anti-41BB antibody to the cultures preferentially increased total TIL outgrowth, particularly that of CD8^+^ TIL. The success rate of TIL growth was increased from 23% to 50% for cultures grown without and with anti-41BB respectively. Sequencing of the T cell receptor CDR3-beta chain found specific T cell clones enriched at the tumor site in comparison to the blood. IHC staining for MHC class I (MHCI) on PDAC tumor samples showed that it is widely expressed but at low levels generally.

**Conclusions**

In conclusion, it is possible to expand CD8^+^ T cells from PDAC bearing TCR sequences highly enriched in the tumor. Additionally, expanded TIL would be able to target tumor cells as they are shown to express MHCI. Although there are barriers yet to overcome, the initial data suggest the feasibility of TIL therapy for PDAC.

### P3 Synthetic biology approaches to enhance adoptive cell therapy safety and precision

#### Scott M Coyle^1^, Kole T Roybel^1^, Levi J Rupp^1^, Stephen P Santoro^1^, Stephanie Secrest^1^, Michael Spelman^1^, Hanson Ho^1^, Tina Gomes^1^, Tiffany Tse^1^, Chia Yung-Wu^2^, Jack Taunton^3^, Wendell Lim^3^, Peter Emtage^1^

##### ^1^Cell Design Labs, San Francisco, CA, USA; ^2^Amgen, San Francisco, CA, USA; ^3^University of California San Francisco, San Francisco, CA, USA

###### **Correspondence:** Stephen P Santoro (steve@celldesignlabs.com)

**Background**

Chimeric antigen receptor T cells (CAR-T) have shown impressive efficacy against numerous hematological malignancies, yet a high percentage of individuals receiving these therapies experience toxicity in the form of cytokine release syndrome (CRS) and/or normal tissue destruction. Furthermore, solid tumors represent a substantive challenge for CAR therapy due to a lack of tumor- specific antigens and general inability of T cells to overcome immunosuppressive tumor microenvironments. We have sought to circumvent these obstacles by utilizing synthetic biology approaches to augment CAR-T function and specificity.

**Methods**

We developed two platform technologies that aim to mitigate toxicity associated with CAR-T therapy and endow T cells with environmental sensing capabilities that enhance tumor discrimination from normal tissue and/or confer the ability to generate customizable response outputs. Firstly, we engineered an “ON-switch” CAR that consists of two protein modules that undergo heterodimerization and become competent for signaling only in the presence of a small-molecule dimerizing agent [1]. In addition, we created a “synthetic Notch” (synNotch) receptor, which we previously described in the context of combinatorial antigen sensing [2], that is capable of driving expression of any number of downstream polypeptides in response to antigen engagement.

**Results**

Here we describe advances in our ON-switch CAR design that allow for dose-dependent antigen-specific T cell activation in the presence of an FDA-approved non-immunosuppressive small molecule dimerizer. Furthermore, we demonstrate that our synNotch T cells are able to deliver therapeutic payloads capable of, but not limited to, modulating the tumor microenvironment and changing the cell-intrinsic transcriptional properties of the synNotch T cells.

**Conclusions**

We have successfully identified a heterodimerizing switch receptor that specifically activates T cells in the presence of an FDA-approved non-immunosuppressive small molecule. The ability to control the potency of the CAR-mediated immune response in this way may reduce the toxicity associated with CAR-T therapy. Furthermore, we have demonstrated that synNotch T cells are able to sculpt the anti-tumor immune response in both a T cell intrinsic (transcriptional programing) and T cell extrinsic (therapeutic payload) manner, providing a customizable platform for altering T cell function and the tumor microenvironment.

**References**

1. Wu C-Y, Roybal KT, Puchner EM, Onuffer J, Lim, WA: **Remote control of therapeutic T cells through a small molecule-gated chimeric receptor.** *Science* 2015 **350**: aab4077.

2. Roybal KT, Rupp LJ, Morsut L, Walker WJ, McNally KA, Park JS, Lim W: **Precision Tumor Recognition by T Cells With Combinatorial Antigen-Sensing Circuits.** *Cell* **164**: 770–779.

## Biomarkers and Immune Monitoring

### P4 Evaluation of anticancer immunity in patients with thyroid cancer with a focus towards developing effective combination immunotherapy

#### Tarsem Moudgil^1^, Carmen Ballesteros-Merino^1^, Traci Hilton^2^, Christopher Paustian^2^, Rom Leidner^3^, David Page^4^, Walter Urba^4^, Bernard Fox^1^, Bryan Bell^3^, Ashish Patel^1^

##### ^1^Providence Portland Medical Center, Portland, OR, USA; ^2^UbiVac, Portland, OR, USA; ^3^Earle A. Chiles Research Institute, Robert W. Franz Cancer Center, Providence Portland Medical Center, Portland, OR, Portland, OR, USA; ^4^Earle A. Chiles Research Institute, Providence Cancer Center, Portland, OR, USA

###### **Correspondence:** Bernard Fox (foxb@foxlab.com)

**Background**

Thyroid cancer is the most common endocrine-related cancer with 64,330 diagnoses expected this year. While the majority of these cancers are curable, almost 2% of these cancers are anaplastic thyroid cancers, which are highly aggressive and almost uniformly lethal. At the same time, the thyroid is known for being inherently immunogenic. For these reasons and due to an active surgical practice providing regular resections of thyroid cancers, we undertook a study of thyroid cancer with the idea of developing an immunotherapy for this disease.

**Methods**

We have developed a thyroid cancer tumor bank to complement our Oral, Head and Neck Cancer Program. This tumor bank cryopreserves enzymatically isolated viable cells from resected tumors (n = 16).We are also attempting to develop primary cell lines and are isolating and assessing autologous tumor-specific functions of tumor-infiltrating lymphocytes (TIL) (n = 7).

**Results**

To date we have established 3 tumor cell lines from thyroid cancer specimens and identified PD-L1 expression on 2 of 2 tested. While numbers are small, preliminary analyses suggest that TIL cultures can be generated from 85% of thyroid cancer specimens and that autologous tumor-reactive TIL can be detected in 43% (n = 7) of thyroid cancers. Since not every tumor appears to contain TIL capable of recognizing autologous tumor, strategies to prime tumor-specific T cells represents an area of interest. DPV-003 is a microvesicle vaccine, DRibble, that contains more than 80 proteins that are overexpressed by thyroid cancer (TCGA provisional RNASeq n = 509 pts). The vaccine also contains a number of DAMPs and agonist activity for multiple TLRs packed into stable double membrane microvesicles that are targeted to CLEC9A+ antigen presenting cells. We are also developing a second thyroid-specific DRibble vaccine from a cell line derived from an anaplastic thyroid cancer.

**Conclusions**

Almost half of thyroid cancers evaluated, including one anaplastic thyroid cancer, contain T cells capable of recognizing autologous cancer cells and secreting IFN-g. However, the other 50% of thyroid cancers appear to lack tumor-reactive T cells and may benefit from combination immunotherapy strategies that include a vaccine.

**Acknowledgements**

Support: Steve and Cindy Harder, Robert W. and Elsie Franz, Wes and Nancy Lematta, Lynn and Jack Loacker, and The Chiles foundation (BAF).

### P5 Development and clinical translation of 89Zr-Df-IAB22M2C for detecting CD8+ T Cells for immunotherapy applications

#### Tove Olafsen^1^, Daulet Satpayev^2^, Michael Torgov^1^, Filippo Marchioni^1^, Jason Romero^1^, Ziyue Karen Jiang^1^, Charles Zamilpa^1^, Jennifer S Keppler^1^, Alessandro Mascioni^1^, Fang Jia^1^, Chen-Yu Lee^1^, Jean Gudas^1^

##### ^1^ImaginAb Inc., Inglewood, CA, USA; ^2^AdicetBio, Inc., Menlo Park, CA, USA

###### **Correspondence:** Jean Gudas (jgudas@imaginab.com)

**Background**

Immunotherapies are changing the landscape for cancer treatment; however, the field is hampered by the lack of biomarkers that can be used for patient selection and for monitoring treatment responses rapidly and non-invasively. To address this need, ImaginAb is developing ^89^Zr-Df-IAB22M2C, an ~80 kDa minibody (Mb) with high affinity to the CD8 glycoprotein (binding EC_50_ = 0.4 nM) conjugated with desferrioxamine (Df) and radiolabeled with the positron emitting radionuclide Zirconium-89 (^89^Zr; T_1/2_ 78.4 hours) for imaging CD8+ T cells in humans.

**Methods**

A comprehensive preclinical program that included evaluation of the *in vitro* and *in vivo* pharmacodynamics of IAB22M2C (unconjugated Mb), Df-IAB22M2C (conjugated Mb intermediate), Zr-Df-IAB22M2C (Zr chelated, conjugated non-radiolabeled form of final drug) and ^89^Zr-Df-IAB22M2C (radioactive final drug product) was conducted to demonstrate the safety and potential efficacy of the probe.

**Results**

*In vitro* studies using human PBMCs from 10 individual human donors showed no measurable or reproducible impact on proliferation, activation or depletion of CD8+ T cells and no consistent release of cytokines when donor CD8+ T cells were exposed to soluble or immobilized Mb protein. Studies that evaluated the effect of saturating concentrations of ^89^Zr-Df-IAB22M2C on proliferation and viability of CD8+ T cells *in vitro,* also showed no impact on these parameters. Preclinical imaging and biodistribution studies demonstrated favorable pharmacokinetics and the ability of ^89^Zr-Df-IAB22M2C to detect infiltrating CD8+ T cells in a mouse hu-PBMC NSG™ GvHD model and in Matrigel^TM^ plugs implanted with different numbers of human CD8+ T cells. Radiation dosimetry studies conducted in hu-CD34 NSG^TM^ mice and the results GLP dosimetry analysis showed that on average, the organs receiving the largest dose equivalent were the kidneys at 8.0 rem/mCi (2.2 mSv/MBq) followed by the liver at 7.9 rem/mCi (2.1 mSv/MBq) and LLI wall at 6.5 rem/mCi (1.8 mSv/MBq). A GLP toxicology study was conducted in cynomolgus monkeys that included multiple dose cohorts of Zr-Df-IAB22M2C and a vehicle control. The results showed that doses up to 25 mg/kg of Zr-Df-IAB22M2C administered weekly to cynomolgus monkeys did not result in any treatment-related findings in survival, clinical signs, body weights, food consumption, ophthalmic examinations, electro-cardiology, blood pressure, heart rate, clinical and anatomic pathology, peripheral blood lymphocyte population, and cytokine levels.

**Conclusions**

^89^Zr-Df-IAB22M2C has the desired sensitivity and safety profile for imaging CD8+ T cells and the first-in-human studies will commence in the Q4 2016.

### P6 High dose interleukin- 2 (HD IL-2) select trial in melanoma: a tissue and blood collection protocol to identify predictive biomarkers of benefit to HD IL-2 in patients with advanced melanoma

#### Ryan J Sullivan^1^, Yujin Hoshida^2^, Theodore Logan^3^, Nikhil Khushalani^4^, Anita Giobbie-Hurder^5^, Kim Margolin^6^, Joanna Roder^7^, Rupal Bhatt^8^, Henry Koon^9^, Thomas Olencki^10^, Thomas Hutson^11^, Brendan Curti^12^, Shauna Blackmon^13^, James W Mier^8^, Igor Puzanov^14^, Heinrich Roder^7^, John Stewart^15^, Asim Amin^16^, Marc S Ernstoff^14^, Joseph I Clark^17^, Michael B Atkins^18^, Howard L Kaufman^19^, Jeffrey Sosman^20^, Sabina Signoretti^8^, David F McDermott^8^

##### ^1^Medical Oncology Department, Massachusetts General Hospital, Boston, MA, USA; ^2^Hess Center for Science and Medicine; Tisch Cancer Institute, New York, NY, USA; ^3^Simon Cancer Center, Indiana University, Indianapolis, IN, USA; ^4^H. Lee Moffitt Cancer Center, Tampa, FL, USA; ^5^Department of Biostatistics & Computational Biology, Boston, MA, USA; ^6^Department of Medical Oncology, City Of Hope, Duarte, CA, USA ^7^Biodesix, Inc., Boulder, CO, USA; ^8^Beth Israel Deaconess Medical Center, Boston, MA, USA ^9^Case Western Reserve University, Cleveland, OH, USA; ^10^The Ohio State University, Columbus, OH, USA; ^11^Texas Oncology-Baylor Charles A. Sammons Cancer Center, Dallas, TX, USA; ^12^Earle A. Chiles Research Institute, Providence Cancer Center, Portland, OR, USA; ^13^Massachusetts General Hospital Cancer Center, Boston, MA, USA; ^14^Roswell Park Cancer Institute, Buffalo, NY, USA; ^15^Wake Forest Baptist Medical Center, Winston Salem, NC, USA; ^16^Levine Cancer Institute, Carolinas HealthCare System, Charlotte, NC, USA; ^17^Loyola University Medical Center, Maywood, IL, USA; ^18^Georgetown-Lombardi Comprehensive Cancer Center, Washington , DC, USA; ^19^Rutgers Cancer Institute of New Jersey, New Brunswick, NJ, USA; ^20^Robert Lurie Comprehensive Cancer Center of Northwestern University, Chicago, IL, USA

###### **Correspondence:** Ryan J Sullivan (rsullivan7@mgh.harvard.edu)

**Background**

HD IL-2 provides objective responses in 15-20% and durable complete remission in 5-8% of patients with metastatic melanoma (MM). We previously identified a gene expression-based tumor subclass characterized by immune related genes (Class 2; C2) associated with durable response to HD IL-2 compared to the remaining tumors that overexpressed lineage-associated genes (Class 1; C1). The primary objective of the HD IL-2 select trial in melanoma was to prospectively validate the favorable gene expression signature (C2). Secondary objectives were to seek serum and tissue biomarkers of durable response.

**Methods**

170 patients with MM were enrolled at 15 Cytokine Working Group sites from 2010 to 2014. All patients had formalin-fixed paraffin-embedded (FFPE) tumor tissues identified and blood drawn prior to HD IL-2. Tumor assessments used WHO criteria and investigator-assessed outcomes. RNA extracted from FFPE tumor tissues was used for whole transcriptome profiling by RNA sequencing (114 samples yielded sufficient RNA, 101 passed default Quality Control (QC)). Pretreatment serum from 114 patients served as the test set and was analyzed using matrix-assisted laser desorption/ionization (MALDI) and machine-based learning algorithms to identify a predictive protein expression signature.

**Results**

Thirty-one of 170 pts (18.2%) responded, and median overall survival was 21.3 months, with a 40 month median follow-up. Analysis of RNAseq from 101 patients whose specimens passed QC showed that a C2 signature was associated with response to HD IL-2 (normalized enrichment score 1.70, false discovery rate 0.004). Using MALDI, a protein expression signature enriched for acute phase proteins (including CRP, IL-6, and SAA) was defined in the pre-treatment serum and used to classify 39 patients into group A (non-acute phase protein expression) and 75 patients in group B (acute phase protein expression). Complete response rate in group A was 21% and zero in group B (p = 0.0001). Two-year PFS rate was 29% in group A compared to 4% in group B (p = 0.0005).

**Conclusions**

In this prospective biomarker validation study, HD IL-2 produced durable remissions and prolonged survival in patients with MM. A tumor-derived gene expression signature enriched for immune-related genes was associated with response. Additionally, preliminary data with a serum protein signature appears to identify patients most likely to have a complete response.

**Trial Registration**

ClinicalTrials.gov identifier NCT01288963.

### P7 Pharmacodynamic gene expression changes from talimogene laherparepvec (T-VEC) plus ipilimumab in a phase Ib study for metastatic melanoma

#### Abraham A Anderson^1*^, Igor Puzanov^2^, Mohammed M Milhem^3^, Robert HI Andtbacka^4^, David Minor^5^, Kevin S Gorski^1^, Daniel M Baker^1^, Omid Hamid^6^, Howard L Kaufman^7^

##### ^1^Amgen Inc., Thousand Oaks, CA, USA; ^2^Roswell Park Cancer Institute, Buffalo, NY, USA; ^3^University of Iowa Hospitals and Clinics, Iowa City, IA, USA; ^4^University of Utah, Huntsman Cancer Institute, Salt Lake City, UT, USA; ^5^California Pacific Melanoma Center, San Francisco, CA, USA; ^6^The Angeles Clinic & Research Institute, Los Angeles, CA, USA; ^7^Rutgers Cancer Institute of New Jersey, New Brunswick, NJ, USA

###### **Correspondence:** Abraham A Anderson (andersoa@amgen.com)

**Background**

T-VEC is a herpes simplex virus type-1 based oncolytic immunotherapy designed to selectively replicate in tumors, produce GM-CSF, and stimulate antitumor immune responses. Ipilimumab is a checkpoint inhibitor that promotes T cell activation by blocking negative signaling through CTLA-4. Both agents have demonstrated activity as monotherapy in advanced melanoma. Based on the potential complementary MOA of the agents, tumor cell lysis and antigen presentation (T-VEC) in combination with T cell checkpoint inhibition, we hypothesized that improved efficacy was possible when the agents are used in combination. Because the safety profiles are non-overlapping, the combination was not anticipated to have significant increased toxicity. To address these hypotheses, a phase Ib/II study evaluating the safety and efficacy of T-VEC plus ipilimumab for Stage III-IV metastatic melanoma was initiated. The phase Ib study was completed (N = 19) with no DLTs (primary endpoint) or new safety signals with combination treatment, and an ORR of 50% [1]. Phase Ib also included biomarker analyses investigating potential pharmacodynamic markers for T-VEC monotherapy and in combination with ipilimumab.

**Methods**

Nineteen patients received T-VEC at 10^6^ PFU/mL at week 1, then 10^8^ PFU/mL Q2W from week 4. Ipilimumab was given at 3 mg/kg Q3W starting at week 6 for 4 infusions. Peripheral blood was obtained (Paxgene RNA) at baseline and at weeks 4, 6, 9, and 15. Gene expression (Agilent Microarray) was analyzed for changes in expression level with treatment. Pharmacodynamic markers were identified with a linear mixed effects model. False discovery was controlled with permutation testing.

**Results**

Gene expression was measured in 16 patients in phase Ib. Most treatment effects on expression were seen after ipilimumab treatment, but there were a few effects in the initial T-VEC phase that passed false discovery controls. These T-VEC effects included *SELV*, *SYNPO*, *ZBTB32*, *IQCF2*, *CDC27*, *KLK1*, *PRR20B*, *CHST6*, and *IGH. ZBTB32* has been reported to control the proliferative burst of virus-specific natural killer cells responding to infection. The combination effects were enriched for genes involved in lymphoid tissue structure and development and immune cell trafficking. 185 of these genes had signs of a T-VEC effect in the monotherapy phase. These included increases in *GZMM*, *PDCD1*, *CD8B*, *CD8A*, and *CTLA4* and decreases in *IL18*, *IRAK3*, and *TXNRD1*.

**Conclusions**

This hypothesis-generating microarray analysis identified genes upregulated in circulating peripheral blood cells after T-VEC monotherapy and combination treatment. We plan to further evaluate these genes and other potential pharmacodynamic markers in phase II.

**Trial Registration**

ClinicalTrials.gov identifier NCT01740297.

**References**

1. Puzanov I, Milhem MM, Minor D, Hamid O, Li A, Chen L, *et al:* **Talimogene laherparepvec in combination with ipilimumab in previously untreated, unresectable stage IIIB-IV melanoma.** *J Clin Oncol* 2016, **34**:2619–2626.

## Clinical Trials in Progress

### P8 Phase I study of alpha-tocopherlyoxyacetic acid in patients with advanced cancer: immune response and pharmacokinetics results

#### Emmanuel Akporiaye^1^, Brendan Curti^2^, Yoshinobu Koguchi^2^, Rom Leidner^3^, Kim Sutcliffe^3^, Kristie Conder^3^, Walter Urba^2^

##### ^1^Veana Therapeutics Inc, Portland, OR, USA; ^2^Earle A. Chiles Research Institute, Providence Cancer Center, Portland, OR, USA; ^3^Earle A. Chiles Research Institute, Robert W. Franz Cancer Center, Providence Portland Medical Center, Portland, OR, USA

###### **Correspondence:** Emmanuel Akporiaye (etakporiaye@gmail.com)

**Background**

Alpha-tocopheryloxyacetic acid (α-TEA) targets tumor cell mitochondria to release reactive oxygen species (ROS) that induce immunogenic cell death (ICD), antigen release, and enhanced antigen cross-presentation in pre-clinical models. α-TEA is being evaluated for safety and tolerability in a first-in-human phase I trial in patients with advanced cancers (NCT02192346). Tumor types in the ongoing trial include renal cancer, esophageal adenocarcinoma, thyroid cancer, duodenal cancer, and squamous cell carcinoma of the head and neck.

**Methods**

α-TEA lysine salt is administered orally to patients and given daily in escalating doses for 28 days. Immune monitoring of peripheral whole blood was conducted for all 12 patients at baseline, and at 1 week and 4 weeks post-treatment. Plasma levels of α-TEA have been determined so far in patients receiving 2.4 mg/kg and 4.8 mg/kg α-TEA at 1, 4, 8, and 24 hours after the first dose. Additional samples were evaluated on days 8, 15, 22, and 29 before the planned α-TEA dose on those days.

**Results**

Twelve patients have been treated so far at 2.4 mg/kg and 4.8 mg/kg dose levels. Eight patients have stable disease, lasting from 1 to 22+ months. One patient showed more than a 2-fold increase in the number of activated (CD38+ HLA-DR+) effector CD8+ T cells 1 week post-treatment. A second patient showed more than a 2-fold increase in the number of activated effector memory CD8+ T cells 4 weeks post-treatment. Both patients experienced stable disease over 5 and 22 months, respectively. Evaluation of α-TEA levels at the 2.4 mg/kg and 4.8 mg/kg doses revealed a proportional increase in α-TEA plasma levels over a 28-day interval without any indication that steady state plasma levels were reached. Of the 12 patients, 6 developed atrial fibrillation (AF) after starting α-TEA. The earliest event occurred 7 days post-treatment, but AF was more common 29-56 days post-treatment. Four of the 6 patients had a medical history of AF. These were grade 2 events by CTCAE 4.0 criteria and managed with appropriate medication without further sequelae.

**Conclusions**

α-TEA treatment resulted in stable disease in 80% of patients lasting between 1 and 22+ months. AF was observed commonly in patients with a medical history of AF, and was managed with appropriate medication. No clinically meaningful grade 3 or 4 toxicities were observed. Plasma α-TEA levels increased proportionally without any indication that steady state levels were achieved. α-TEA may function through enhancing pre-existing CD8+ T cell-mediated anti-tumor activity.

### P9 Phase I, first-in-human, open label, dose escalation study of MGD009, a humanized B7-H3 x CD3 dual-affinity re-targeting (DART®) protein in patients with B7-H3-expressing neoplasms or B7-H3 expressing tumor vasculature

#### Anthony W Tolcher^1^, Evan W Alley^2^, Gurunadh Chichili^3^, Jan E Baughman^4^, Paul Moore^3^, Ezio Bonvini^3^, Syd Johnson^3^, Sadhna Shankar^3^, James Vasselli^3^, Jon Wigginton^3^, John Powderly^5^

##### ^1^START - South Texas Accelerated Research Therapeutics, LLC, San Antonio, TX, USA; ^2^Penn Presbyterian Medical Center, University of Pennsylvania, Philadelphia, PA, USA; ^3^MacroGenics, Inc., Rockville, MD, USA; ^4^MacroGenics, Inc., South San Francisco, CA, USA; ^5^Carolina BioOncology Institute, PLLC, Huntersville, NC, USA

###### **Correspondence:** Jan E Baughman (baughmanj@macrogenics.com)

**Background**

MGD009 is a B7-H3 x CD3 dual affinity re-targeting (DART) protein. DART proteins are bispecific, antibody-based molecules that can bind two distinct antigens simultaneously. MGD009 is designed to redirect T cells to eliminate B7-H3-expressing target cells through co-engagement of B7-H3 on the target cell and CD3 on the T cell, each in a monovalent binding manner. MGD009 contains a mutated human IgG1 Fc domain to reduce/eliminate binding to Fc gamma receptors (FcγRs) and complement, but retains binding to the neonatal Fc receptor (FcRn) enabling use of the IgG salvage pathway to prolong its circulating half-life. B7-H3 protein expression is limited in normal human tissues, but is overexpressed in a broad range of tumor types, including melanoma, non-small cell lung cancer (NSCLC), squamous cell carcinoma of the head and neck (SCCHN), mesothelioma, and urothelial cancer. By binding to B7-H3 on tumor cells and CD3 on T lymphocytes, MGD009 can recruit cytotoxic T cells, irrespective of their ability to recognize cancer cells, and trigger T cell activation, expansion, and T cell-mediated killing of B7-H3-expressing tumors. The limited expression of B7-H3 on normal cells should restrict the potential for cytolytic activity directed at normal tissues, and unintended toxicity in patients treated with MGD009.

**Methods**

This multi-center, open-label trial is a phase I dose escalation/cohort expansion study. All patients must have advanced B7-H3-positive tumors. Prior checkpoint inhibitor therapy will be allowed. Dose escalation uses a 3+3+3 design, with patients treated every 2 weeks with escalating doses of IV MGD009 (starting dose 0.3 ug/kg). The dose escalation phase enrolls patients with mesothelioma, bladder cancer, melanoma, SCCHN, NSCLC, clear cell renal cell carcinoma, ovarian cancer, thyroid cancer, triple-negative breast cancer, pancreatic cancer, colon cancer, soft tissue sarcoma, or prostate cancer. Cohort expansions (n=16/cohort) treated at the maximum tolerated dose will include patients with melanoma, SCCHN, mesothelioma, urothelial cancer, and NSCLC. Pre- and on-study biopsies are required for melanoma patients in the cohort expansion phase. Primary and secondary study objectives include characterization of the safety, pharmacokinetics, pharmacodynamics, and preliminary antitumor activity of MGD009. All tumor evaluations will be carried out by both Response Evaluation Criteria in Solid Tumors (RECIST 1.1) and immune-related RECIST.

**Trial Registration**

ClinicalTrials.gov identifier NCT02628535.

### P10 Intratumoral Flt3L and poly-ICLC combined with low dose radiotherapy: a novel in situ vaccine for incurable indolent lymphomas

#### Thomas Marron^1^, Nina Bhardwaj^1^, Linda Hammerich^1^, Fiby George^1^, Seunghee Kim-Schulze^1^, Tibor Keler^2^, Tom Davis^2^, Elizabeth Crowley^2^, Andres Salazar^3^, Joshua Brody^4^

##### ^1^Icahn School of Medicine at Mount Sinai, New York, NY, USA; ^2^Celldex Therapeutics, Hampton, NJ, USA ^3^Oncovir, Inc., Washington, DC, USA; ^4^Mount Sinai School of Medicine, New York, NY, USA

###### **Correspondence:** Thomas Marron (thomas.marron@mountsinai.org)

**Background**

Lymphomas are the 5^th^ most common cancer in the United States, and unlike more aggressive lymphomas, indolent non-Hodgkin lymphoma (iNHL) and is incurable with standard therapy. A previous trial of *in situ* vaccination in iNHL combined intratumoral injection of a TLR9 ligand with radiation to induce a systemic immune response against tumor. This approach induced tumor-specific CD8+ T cell responses and durable clinical remissions of patients’ untreated sites of disease were seen in a minority of patients. One limitation in this previous trial may have been the scarcity of intratumoral dendritic cells (DCs); DCs are uniquely able to endocytose dying tumor cells for cross-presentation to tumor antigen-specific CD8+ T cells. In our novel iteration of *in situ* vaccine, intratumoral FMS-like tyrosine kinase 3 ligand (Flt3L) is introduced as a priming step to increase the presence of intratumoral DCs. Flt3L induced tumor leukocyte infiltration and regression in lymphoma tumors in pre-clinical trials, and CDX-301- a formulation of Flt3L - was previously shown to mobilize BDCA-1 and BDCA-3 myeloid DC subsets in an early phase clinical trials. These DC subsets respond to several TLR agonists and cross-present antigens more effectively than plasmacytoid DCs (pDCs). While pDCs are high expressers of TLR9, responsive to CpG, myeloid dendritic cells express a wider array of TLRs, including high levels of TLR3.

**Methods**

This phase I/II trial tests the hypothesis that this novel *in situ* vaccination will induce clinical remissions at distant (untreated) tumor sites in two cohorts of patients with either previously untreated or relapsed/refractory iNHL (n = 15 per group). Intratumoral CDX-301 25ug/kg is injected into a palpable lymph node for 9 days, followed by 2Gy local radiotherapy on day 9 and 10 to the target lymph node. To activate local DCs, poly-ICLC 2 mg is injected on day 10, 14, 17, and weekly thereafter for a total of 8 treatments.

**Results**

Exploratory endpoints include measuring induction of systemic tumor-specific immune response in pre- and post-vaccine blood and tissue samples. Using flow cytometry and CyTOF, we have confirmed that CD1c + (BDCA1^+^) and CD141+ (BDCA3^+^) DCs home to treated tumors following treatment with Flt3L and T cells attain a mature effector phenotype. Tissue from initial bx is being sequenced, and candidate neoantigens being determined *in silico*; these neoantigens are then being synthesized and tested for potential to activate patient pre- and post-vaccination T cells.

**Conclusions**

This trial is in process.

**Trial Registration**

ClinicalTrials.gov identifier NCT01976585.

### P11 Preliminary safety and efficacy data for radiotherapy and PD-L1 checkpoint blockade in metastatic non-small cell lung cancer: is timing everything?

#### Arta Monjazeb^1^, Megan E Daly^2^, Jonathan Riess^2^, Tianhong Li^2^, William J Murphy^1^, Karen Kelly^2^

##### ^1^University of California, Davis, Sacramento, CA, USA; ^2^University of California Davis Comprehensive Cancer Center, Sacramento, CA, USA

###### **Correspondence:** Arta Monjazeb (ammonjazeb@ucdavis.edu)

**Background**

Inhibition of the PD-1/PD-L1 checkpoint pathway can induce rapid and durable responses in patients with non-small cell lung cancer (NSCLC). Unfortunately the majority of patients fail to respond and there is interest in exploring combinatorial strategies to improve response rates. One such strategy is combining checkpoint inhibition with radiotherapy (RT). We report here our pre-clinical data for combining radiotherapy with PD-L1 checkpoint blockade. These data demonstrate a clear influence of the sequencing of combinatorial therapy on its efficacy. Based on these data, we have initiated a clinical trial testing sequencing strategies of radiotherapy with PD-L1 inhibition in patients with metastatic NSCLC.

**Methods**

Using syngeneic mouse tumor models, we tested the synergy of combining RT with PD-L1 inhibition and the influence of the sequencing of these therapies on efficacy. Based on this preclinical work, we have initiated a phase II clinical trial testing this combinatorial strategy with three cohorts. The three cohorts are concurrent therapy, radiotherapy followed by PD-L1 checkpoint blockade, and PD-L1 blockade followed by radiotherapy. We report here the preliminary safety, efficacy, and correlative science data from interim analysis of the safety run-in for this trial.

**Results**

In studies using syngeneic mouse tumor models, we find that PD-L1 inhibition provides no added benefit to radiotherapy alone when administered after radiotherapy. Conversely, priming of the immune system with anti-PD-L1 prior to RT provides significant synergy of the combinatorial therapy. Our clinical trial has completed enrollment to the safety-run in of 6 patients. In total, 2 patients experienced grade 3 dose limiting toxicities meeting the criteria for completion of the safety-run in without the need for dose de-escalation. One patient experienced asymptomatic grade 3 lymphopenia and one patient experienced both grade 3 lymphopenia and grade 3 failure to thrive. At twelve weeks post-treatment initiation, 83% of patients experienced response or disease stability. Three patients (50%) had abscopal responses by RECIST criteria, two patients (33%) had stable disease, and one patient (17%) had progressive disease.

**Conclusions**

Pre-clinical data suggests that sequencing may be key to the success of combinatorial strategies of PD-L1 blockade and RT. The safety run-in for combining PD-L1 checkpoint inhibition with RT suggests that the combination is safe and tolerable in metastatic NSCLC. The trial will continue to accrual to evaluate different sequencing strategies for combining RT and PD-L1 checkpoint blockade. Further study is needed to evaluate the efficacy and optimal sequencing of RT + PD-L1 checkpoint blockade.

## Combinations: Immunotherapy/Immunotherapy

### P12 Tissue factor is a novel oncotarget in triple negative breast cancer and BRAF- mutated melanoma for immunotherapy using a second generation ICON (L-ICON) in monotherapy and combination therapy

#### Zhiwei Hu^1^, Rulong Shen^2^, Amanda Campbell^2^, Elizabeth McMichael^2^, Lianbo Yu^2^, Bhuvaneswari Ramaswam^2^, Cheryl A London^2^, Tian Xu^3^, William Carson^2^

##### ^1^The Ohio State University Wexner Medical Center and The OSU James Comprehensive Cancer Center, Columbus, OH, USA; ^2^The Ohio State University, Columbus, OH, USA; ^3^Yale University, New Haven, CT, USA

###### **Correspondence:** Zhiwei Hu (zhiwei.hu@osumc.edu)

**Background**

The objective of this study is to identify tissue factor (TF) as a novel oncotarget for triple negative breast cancer (TNBC) and BRAF mutated melanoma, both of which are very difficult to treat in clinic, and to develop a novel TF-targeting agent for immunotherapy. To achieve this goal, Hu developed a second generation TF-targeting ICON, named L-ICON, which consists of only the light chain (1-152 aa) of FVII fused to an IgG1Fc. The effects of L-ICON were evaluated as monotherapy or combination therapy with interleukin 15 (IL-15) for the malignancies.

**Methods**

TF expression was determined by immunohistochemistry or by flow cytometry. L-ICON protein (GenBank accession no. KY760097) and replication-deficient adenoviral vectors have been developed. Binding activity of L-ICON was determined. Its ADCC effect was evaluated by an ADCC effector assay and coagulation activity by FVII chromogenic activity assay. L-ICON efficacy in monotherapy and combination therapy with IL-15 was tested in mouse models of murine and human breast cancer (4 T1 and TNBC MDA-MB-231) and melanoma (B16F10 and BRAF mutated SK-Mel-28).

**Results**

TF is over-expressed on TNBC cells and the tumor neovasculature in over 85% of TNBC patients (n = 14) when using standard paraffin-embedded tumor tissues or in nearly 60% of TNBC patients (n = 157) when employing tissue microarray slides. Importantly, TF expression is not detected in normal breast tissues. L-ICON has several important improvements over its first generation ICON, including (i) more than 50% reduction in molecular mass, (ii) complete elimination of coagulation activity, (iii) stronger binding activity to TNBC and (iv) more effective as monotherapy *in vivo* in orthotopic and subcutaneous mouse models of human TNBC (MDA-MB-231) and murine cancer 4 T1 (an animal stage IV human breast cancer) and B16F10. L-ICON monotherapy and combination with IL-15 were effective for the treatment of SK-Mel-28 in SCID mouse models.

**Conclusions**

TF is a novel biomarker and oncotarget in TNBC and BRAF- mutated melanoma. L-ICON, a novel TF-targeting ICON, was effective in monotherapy and combination therapy with IL-15 for the treatment of murine and human TNBC and melanoma *in vitro* and *in vivo* in preclinical mouse models.

**Acknowledgements**

This work was partly supported by a startup fund from OSUMC, a Seed Award from the OSUCCC TT Program, a Phase 1 L-Pilot Award from OSU CCTS via NCATS Award Number UL1TR001070 and the Dr. Ralph and Marian Falk Medical Research Trust. IL-15 was obtained from the NCI Preclinical Repository. Z.H. is the inventor of L-ICON and its uses (US Patent Application # 62/082,891).

### P13 Beta-adrenergic blockade improves the immunotherapeutic response to melanoma

#### Kathleen M Kokolus^1^, Elizabeth A Repasky^2^, Todd D Schell^1^, Joseph D Drabick^1^

##### ^1^The Pennsylvania State University College of Medicine, Hershey, PA, USA; ^2^Roswell Park Cancer Institute, Buffalo, NY, USA

###### **Correspondence:** Kathleen M Kokolus (kokolusk@hmc.psu.edu)

**Background**

Recent developments in immunotherapy have made enormous strides towards expanding the scope of cancer treatment by targeting a patient’s own immune cells. Despite these advances, malignant melanoma remains a significant clinical issue with a high proportion of patients remaining unresponsive to therapy and improved, but still low, complete response rates. The body’s response to stress is closely integrated with the immune response, yet few cancer treatment strategies account for the relationship between these biological systems. When the stress response is activated, neurotransmitters, including norepinephrine, which bind β-adrenergic receptors (βARs) located on the surface of immune cells, are released, leading to regulation of various immune cell functions. βAR signaling can be prevented pharmaceutically with βAR antagonists (β-blockers) and considerable literature suggests that these drugs, which are commonly prescribed for other indications including hypertension, are associated with positive outcomes in cancer patients. We examined the effects of βAR blockade on the efficacy of two immunotherapies approved to treat metastatic melanoma: IL-2, which promotes T cell proliferation and αPD-1, which impacts T cell activation.

**Methods**

C57BL/6 J mice with established B16.F10 melanomas were treated with β-blockers and immunotherapy (αPD-1, IL-2 or αPD-1/IL-2) and tumor growth was monitored throughout each treatment regimen. The accumulation of immune cells within the tumors and lymphoid tissues were evaluated by flow cytometry at multiple time points following treatment.

**Results**

Blockade of βAR signaling beginning after tumors were established had no significant impact on tumor growth. In contrast, attenuation of tumor growth by each immune-based therapy was improved in the presence of β-blockers. We observed significantly extended survival in mice treated with αPD-1 or αPD-1/IL-2 combined with β-blockers compared to immunotherapy only mice. Most importantly, the combination of β-blockers, αPD-1 and IL-2 produced a highly significant delay in tumor growth and prolonged survival compared to αPD-1/IL-2 without β-blockers.

**Conclusions**

Blocking βAR signaling improved the efficacy of at least two types of immunotherapy, but was most effective when administered with dual-immunotherapy. We suggest that each therapeutic component may improve a unique aspect of the immune response to maximally delay melanoma progression. Due to the availability of all three components for use in humans, this therapeutic regimen can potentially be clinically translated to expand the population of metastatic melanoma patients who experience long term benefits from immune-based therapies.

**Acknowledgements**

This work was supported by CURE Grant SAP #4100072562 (Pennsylvania Department of Health) and NIH/NCI 5 T32 CA060395 (KMK). IL-2 was generously provided by Prometheus Laboratories Inc.

### P14 Sequentially targeting upregulated TIM-3 and CTLA-4 does not rescue the attenuated therapeutic efficacy of combination immunotherapy with OX40 costimulation and PD-1 blockade

#### David J Messenheimer^1^, Shawn Jensen^2^, Bernard Fox^2^

##### ^1^Earle A. Chiles Research Institute, Portland, OR, USA; ^2^Providence Cancer Center, Portland, OR, USA

###### **Correspondence:** David J Messenheimer (messenhe@ohsu.edu)

**Background**

Combination immunotherapy targeting checkpoint molecules has shown substantial results against solid tumors. However as novel therapies targeting costimulatory molecules are introduced into the clinic, successful combination with checkpoint blockade remains uncertain. Some strongly immunogenic preclinical models have shown benefit when combining anti-OX40 and anti-PD-1 treatment. In contrast, using a PD-1 refractory mammary tumor model we have demonstrated that a significant anti-tumor effect generated with OX40 costimulation is significantly attenuated with the addition of PD-1 blockade. We noted high levels of inflammatory cytokines in the serum of combination treated mice, and also saw a significant increase in inhibitory receptors TIM-3, LAG-3, and CTLA-4 on CD4^+^ and CD8^+^ T cells in the periphery of treated mice. We hypothesized that the upregulation of these other inhibitory receptors were limiting the efficacy of anti-OX40 plus anti-PD-1 combination treatment, and tested whether sequentially blocking these receptors could augment therapeutic efficacy.

**Methods**

Established orthotopically transplanted MMTV-PyMT mammary tumors in FVB/NJ mice were treated with three doses of anti-OX40 and anti-PD-1 every other day. Serum was taken at time points and tested for cytokine concentration and spleens were taken two days after treatment to measure surface expression of inhibitory and costimulatory receptors. Combination treated mice were then followed by three doses of anti-TIM-3 with or without anti-CTLA-4.

**Results**

The addition of anti-TIM-3 and CTLA-4 provided no additional impact to tumor growth compared to that provided by anti-OX40 and anti-PD-1 (n = 7/group, two independent experiments).

**Conclusions**

These data demonstrate that TIM-3 and CTLA-4 blockade was not sufficient to augment the inhibitory effects of the concurrent combination of anti-OX40 and anti-PD-1, and suggest that targeting inhibitory receptors upregulated after initial immunotherapy treatment will require more sophisticated biomarkers and immune monitoring. Alternatively, we noted significant increases in some costimulatory receptors (ICOS, 4-1BB) and targeting these receptors may provide an alternative to blocking inhibitory pathways. Additionally there may be a tipping point where providing additional antibodies targeting costimulatory or checkpoint molecules may be ineffective. T cells may become so heavily deregulated or over-stimulated that they become permanently exhausted. In support of this we have previously shown that anti-OX40 combined sequentially with anti-PD-1 provides superior therapeutic effect compared to concurrent combination, with complete tumor regression in ~30% of animals treated. Sequentially treated animals generated T cells capable of significantly more long-term proliferation, suggesting that timing is critical when combining immunotherapies.

**Fig. 4 (abstract P14).**
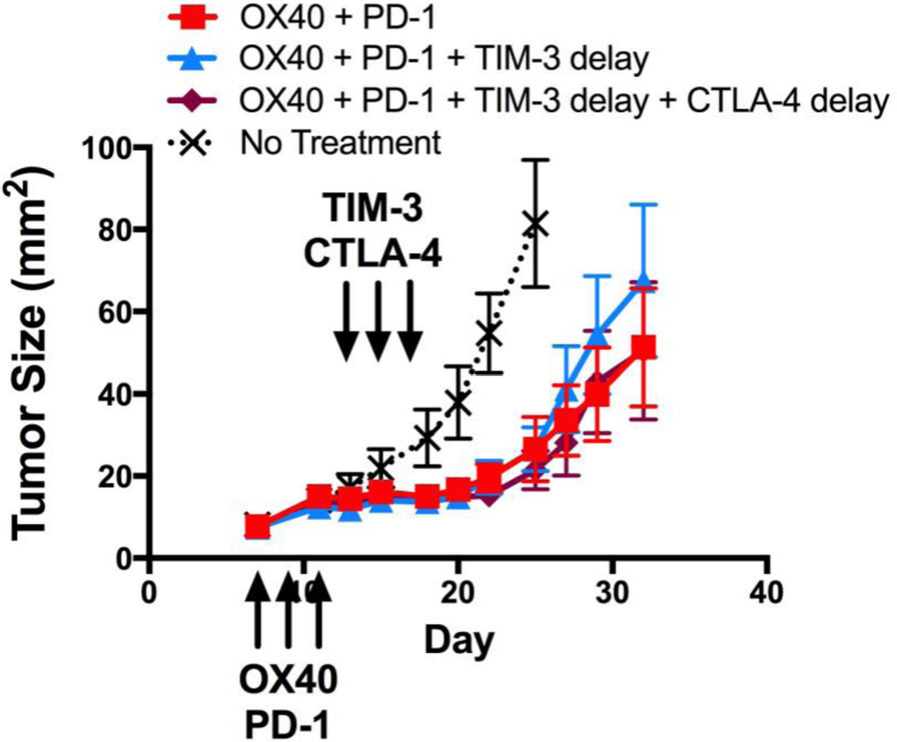
The addition of anti-TIM + anti-CTLA-4 provides no benefit to anti-OX40 + anti-PD-1 combination treatment

### P15 Novel IL-2/mAb complexes mediate potent anti-tumor immunity which is augmented with anti-PD-1 mAb therapy

#### Mark Rubinstein^1^, Kristina Andrijauskaite^1^, Marzena Swiderska-syn^1^, Kristin Lind^2^, Agnes Choppin^2^, Marina K Roell^2^

##### ^1^Medical University of South Carolina, Charleston, SC, USA; ^2^XOMA Corporation, Berkeley, CA, USA

###### **Correspondence:** Mark Rubinstein (rubinsmp@musc.edu)

**Background**

Recent success and FDA approval of immune checkpoint inhibitors (CI) in a growing number of cancers are transforming cancer treatment and revitalizing interest in immunotherapies. However, while efficacy is observed in patients with advanced metastatic diseases treated with CI, not all patients respond and most responses are incomplete. Preclinical studies suggest that combinations of additional modalities will provide opportunities to improve patient responses. As both IL-2 and CI therapy can independently augment anti-tumor immunity in patients, likely in mechanistically distinct ways, we hypothesized we could improve anti-tumor immunity by combining IL-2 and anti-PD-1 mAb therapy.

**Methods**

To improve IL-2 efficacy and therapeutic index, we generated novel anti-IL-2 mAbs which, when complexed with IL-2 (IL-2/mAb) offer advantages over standard IL-2 therapy [1-3]. First, binding to an anti-IL-2 mAb increases IL-2 half-life and biological activity. Second, depending on the epitope at which the mAb binds to IL-2, antibody binding can modulate which IL-2 receptor subunits (alpha, beta, or gamma) are engaged. Antibodies that interfere with binding of IL-2Rα can reduce activation of high- IL-2Rα-expressing cell types, such as suppressive Tregs, and steer activity toward cell types expressing only IL-2Rβ and γ. In this way, these complexes may have more effective anti-tumor activity [1-3]. We screened antibody phage libraries to identify antibodies that shift IL-2 receptor binding and activity differentially on different cell types *in vitro* and *in vivo*. Complexes of these antibodies were tested *in vivo* for their effects on T cell frequency and activation, and in a subcutaneous Lewis lung carcinoma model for their ability to mediate anti-tumor immunity, both alone and in combination with anti-PD-1 mAb.

**Results**

In normal mice, IL-2/mAb complexes potently expanded CD8+ T cells and NK cells with minimal expansion of Tregs. As single agent therapy, IL-2/mAb complexes or anti-PD-1 mAb reduced tumor growth, although most mice succumb to tumor growth eventually. Combination of IL-2/mAb complexes with anti-PD-1 mAb therapy resulted in durable, complete responses in nearly half of the mice.

**Conclusions**

While immune based therapies such as anti-PD-1 mAb can be highly effective in select patients, even in those patients that obtain clinical benefit, disease may recur. Our results suggest that the addition of IL-2/mAb complexes to therapy with anti-PD-1 mAb could broadly increase the percentage of patients deriving benefit from immune-based therapy.

**References**

1. Sato, *et al: Biotherapy* 1993, **6(3)**:225–231.

2. Boyman, *et al: Science* 2006, **311**:1924–1927.

3. Létourneau, *et al: PNAS* 2010, **107**:2171–2176.

### P16 The combination of an IL-15/IL-15Ralpha complex (ALT-803) and anti-PD-1 mAb leads to superior anti-tumor immunity in a murine lung tumor model

#### John Wrangle^1^, Kristina Andrijauskaite^1^, Marzena Swiderska-syn^1^, Peter Rhode^2^, Hing Wong^2^, Mark Rubinstein^1^

##### ^1^Medical University of South Carolina, Charleston, SC, USA; ^2^Altor BioScience Corporation, Miramar, FL, USA

###### **Correspondence:** Mark Rubinstein (markrubinstein@musc.edu)

**Background**

Administration of antibodies that block the PD-1/PD-L1 pathway has demonstrated unprecedented success in mediating clinical responses in patients with advanced cancer. These antibodies are thought to act by blocking the ability of PD-L1 to mediate an inhibitory signal to PD-1 expressing T cells during antigen-recognition. These antibodies are now FDA-approved for multiple cancers including non-small cell lung cancer (NSCLC) in patients with disease that has progressed during or after platinum-based chemotherapy. In these patients, one in five patients can attain a clinical response while on checkpoint inhibitor therapy. While promising, this therapy fails to induce a durable clinical response in most patients. To overcome this limitation, we hypothesized that combinatorial therapy with anti-PD-1 mAb and a lymphocyte growth factor would more effectively augment the expansion of tumor-reactive lymphocytes. This would also provide a means to not only remove inhibitory pathways but directly augment the function of tumor-reactive lymphocytes. We chose to use an IL-15/IL-15Ra complex (ALT-803) composed of an IL-15 mutant (N72D) that was pre-associated with the soluble IL-15Ra/Fc fusion protein. This superagonist complex has been shown to potently expand and activate CD8^+^ T cells and NK cells in various animal models.

**Methods**

To assess the efficacy of combination therapy, we injected C57BL/6 mice subcutaneously with Lewis lung carcinoma. Mice with established tumors were treated with anti-PD-1 mAb and/or IL-15/IL-15Ra complex, and we monitored tumor progression and changes in immune cell populations in the periphery and tumor.

**Results**

The combination of anti-PD-1 mAb and the IL-15/IL-15Ra complex was substantially more effective at inducing complete responses compared with administration of either agent alone. Effective therapy was associated with the expansion of CD8^+^ T cells and NK cells, and the acquisition of the ability of CD8^+^ T cells to produce IFNγ after activation. Interestingly, *in vitro*, IFNγ led to upregulation of both MHC and PD-L1 on tumor cells, suggesting a mechanistic basis for the improved efficacy of the combination therapy.

**Conclusions**

Our results suggest that the efficacy of anti-PD-1 mAb therapy may be improved by co-administration of the IL-15/IL-15Ra complex. Our results also suggest a mechanistic basis for why the combination may be superior to single agent therapy. To determine if this combination would be of value in human patients, we have initiated a phase Ib/II clinical trial (NCT02523469) to assess the combination of anti-PD-1 mAb (nivoluamb) in combination with ALT-803 in patients with refractory advanced NSCLC.

### P17 Functional dichotomy of PI3K isoforms in CD4 T cells provides a strategy for selectively targeting regulatory T cells to enhance anti-tumor immunotherapy

#### Shamim Ahmad^1^, Mason Webb^1^, Rasha Abu-Eid^1^, Rajeev Shrimali^1^, Vivek Verma^1^, Atbin Doroodchi^1^, Zuzana Berrong^1^, David Yashar^1^, Raed Samara^2^, Mikayel Mkrtichyan^1^, Samir Khleif^1^

##### ^1^Georgia Cancer Center, Augusta, GA, USA; ^2^Qiagen, Frederick, MD, USA

###### **Correspondence:** Samir Khleif (skhleif@augusta.edu)

**Background**

The PI3K-Akt signaling pathway modulates diverse biological responses including signaling, proliferation and survival of T cells. The identification of a signaling pathway, which differentially regulates regulatory T cells (Tregs) and conventional T cells (Tconvs), is crucial for selectively modulating these two subsets. The differential role of class IA PI3K isoform in regulating the survival and apoptosis of Tregs and Tconvs has not been elucidated yet.

**Methods**

For *in vitro* experiments sorted Tregs and Tconvs were labeled with CellTrace ^TM^ Violet Cell Proliferation stain (VCT) according to the manufacturer’s protocol (Life Technologies, NY). Cells were stimulated with and without inhibitors. For *in vivo* experiments C57BL/6 Mice were injected subcutaneously (s.c.) with TC-1 tumor cells and monitored for development of tumors. Vaccine was given weekly s.c. For therapeutic experiments vaccine was given weekly throughout the experiment. CAL-101 treatment was provided on the day when tumor size reached 3-4 mm 5-6 day before vaccination.

**Results**

Here, we report that PI3Kd isoform is sufficient for TCR downstream signaling, proliferation, and survival for either Tconvs or Tregs. In Tregs, however, PI3Kd is a dominant isoform, where Tregs are fully dependent on PI3Kd to regulate these properties as PI3Kα and PI3Kb do not play any role in these biologic processes. On the other hand, in Tconvs, the two other isoforms, PI3Kα and PI3Kb combined, provide redundant pathway to PI3Kd in the regulation of TCR signaling, proliferation and survival. This redundant role provided by PI3Kα and PI3Kβ isoforms to PI3Kd in Tconvs offers a selective therapeutic approach to inhibit Tregs, where by inhibiting PI3Kd, signaling, proliferation, and survival are inhibited in Tregs, while PI3Kα and PI3Kβ, will provide a path for Tconvs to proliferate and function.

Importantly, we demonstrate that our findings translate to therapeutic efficacy *in vivo*, where the inhibition of PI3Kδ, enhanced anti-tumor efficacy of antigen-specific vaccine by decreasing the suppressive Tregs and increasing the number of vaccine-induced CD8 T cells, thus showing synergistic therapeutic effect against tumors. Our findings provide a strategy for the selective targeting of Tregs in the frame of cancer combination immunotherapy.

**Conclusions**

These findings provide a new insight into CD4 T cell biology and offer a new strategy for selective targeting of Tregs in the frame of development of anti-cancer immunotherapies.

## Combinations: Immunotherapy/Standard of Care

### P18 Pembrolizumab in combination with chemoradiotherapy (CRT) for locally-advanced squamous cell carcinoma of the head and neck (SCCHN): Interim safety analysis (ISA)

#### Steven Powell^1^, Mark Gitau^2^, Christopher Sumey^1^, Andrew Terrell^2^, Michele Lohr^1^, Ryan K Nowak^1^, Steven McGraw^3^, Ash Jensen^2^, Miran Blanchard^2^, Kathryn A Gold^4^, Ezra EW Cohen^4^, Christie Ellison^1^, Lora Black^1^, John Lee^5^, William Chad Spanos^1^

##### ^1^Sanford Cancer Center, Sioux Falls, SD, USA; ^2^Sanford Roger Maris Cancer Center, Fargo, ND, USA ^3^Sanford Health, Sioux Falls, SD, USA; ^4^Moores Cancer Center, University of California, San Diego, La Jolla, CA, USA; ^5^NantKwest, Inc., Culver City, CA, USA

###### **Correspondence:** Steven Powell (steven.powell@sanfordhealth.org)

**Background**

Blockade of the programmed death receptor 1 (PD-1) interaction with its ligands (PD-L1, PD-L2) represents an active therapeutic approach in recurrent and metastatic SCCHN [1]. This immune checkpoint may allow immune escape during standard treatment, including CRT [2]. Standard CRT in SCCHN has substantial toxicity and the safety of adding PD-1 inhibition is unknown. We report the first ISA of weekly cisplatin-based CRT in combination with pembrolizumab, a humanized IgG4 monoclonal antibody inhibitor of the PD-1:PD-L1/2 interaction.

**Methods**

**Fig. 5 (abstract P18).**
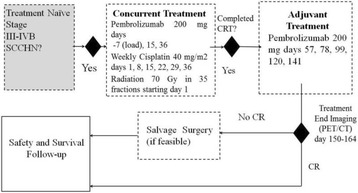
Clinical Trial Schema

**Fig. 6 (abstract P18).**
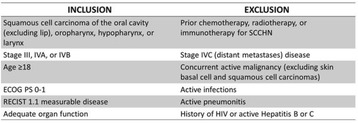
Key Inclusion and Exclusion Criteria

This is an open-label phase IB trial of pembrolizumab used in combination with cisplatin-based, definitive CRT in patents with stage III-IVB SCCHN. The treatment schema is outlined in Fig. 5. Key inclusion and exclusion criteria are in Fig. 6. The primary endpoint of safety is assessed by dose-limiting grade ≥3 adverse events (AEs) and immune-related AEs (irAEs) per NCI-CTCAE v4.0 criteria. Efficacy is measured by RECIST v1.1 complete response (CR) rate on 100-day post-CRT imaging and/or pathologic CR for those who undergo salvage surgery. Planned enrollment is 39 patients based on a hypothesized CR rate of >60% and a two-stage Simon minimax design (type I error rate of 0.05 and power of 0.80). Secondary endpoints will include overall response, survival, and quality-of-life assessments.

**Results**

**Fig. 7 (abstract P18).**
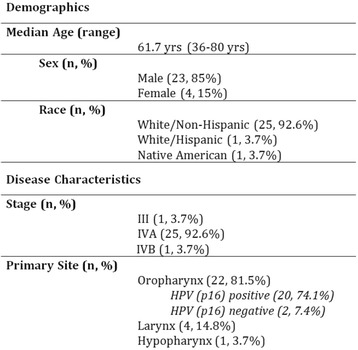
Patient and Disease Characteristics

**Fig. 8 (abstract P18).**
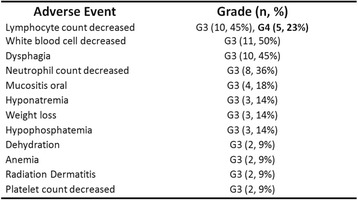
Acute Grade ≥3 Adverse Events with >1 Occurrence

At the time of ISA, 27 patients have been enrolled. Patient and disease characteristics are in Fig. 7. At data cut-off on 9/14/2016, 22 patients have completed CRT. Of those patients, 3 required cisplatin dose reductions and 6 required a dose discontinuation. Acute grade (G) ≥ 3 AEs for those completing CRT are in Fig. 8. Median cumulative cisplatin dose is 225 mg/m2. There were no radiation dose-limiting toxicities. Two patients (9%) discontinued pembrolizumab due to irAEs (G2 peripheral motor neuropathy and G3 transaminase elevation). No deaths have occurred.

**Conclusions**

This represents one of the first studies to evaluate the safety of concurrent CRT and PD-1 inhibition in SCCHN. Acute CRT-related toxicities are comparable to other SCCHN CRT studies. No new immunologic safety signals were seen. Further investigation of this approach is warranted.

**Acknowledgements**

Funding supported by the Merck Investigator Studies Program.

**Trial Registration**

ClinicalTrials.gov identifier: NCT02586207

**References**

1. Seiwert TY, *et al*: **Safety and clinical activity of pembrolizumab for treatment of recurrent or metastatic squamous cell carcinoma of the head and neck (KEYNOTE-012): an open-label, multicentre, phase 1b trial**. *Lancet Oncol* 2016, **17(7)**:956–965.

2. Parikh F, *et al*: **Chemoradiotherapy-induced upregulation of PD-1 antagonizes immunity to HPV-related oropharyngeal cancer**. *Cancer Res* 2014, **74(24)**:7205–7216.

## Diet, Exercise and/or Stress and Impact on the Immune System

### P19 Exercise training reduces splenic accumulation of MDSCs and delays tumor progression in a therapeutic breast cancer model

#### Erik Wennerberg^1^, Emily Schwitzer^2^, Claire Lhuillier^1^, Graeme Koelwyn^3^, Rebecca Hiner^2^, Lee Jones^2^, Sandra Demaria^4^

##### ^1^Weill Cornell Medicine, New York, NY, USA; ^2^Memorial Sloan Kettering Cancer Center, New York, NY, USA; ^3^New York University School of Medicine, New York, NY, USA; ^4^Department of Radiation Oncology, Weill Cornell Medicine, New York, NY, USA

###### **Correspondence:** Erik Wennerberg (erw2010@med.cornell.edu)

**Background**

Epidemiological studies show a correlation between physical activity and cancer-related mortality [1]. However, the contribution of immune mediated anti-tumor immunity to the beneficial effects of exercise has yet to be defined [2]. We sought to investigate if forced running would have a therapeutic benefit in mice bearing a poorly immunogenic breast cancer and investigate the immunological changes occurring in response to exercise.

**Methods**

On day 0 Balb/c mice were inoculated with 4 T1 breast cancer cells subcutaneously in the right flank (n = 6/group). Starting on day 7, once tumors were palpable, mice were subjected to 30 minutes of forced treadmill running (18 cm/sec) five days per week. Control mice remained sedentary throughout the study. Analysis of immune cells in spleen and tumor was performed at day 17 and 32 and spontaneous lung metastases were evaluated at day 32.

**Results**

We observed a significantly delayed primary tumor growth (tumor volume on day 31: 1167 ± 174 mm^3^ in sedentary versus 847 ± 124 mm^3^ in exercised mice, p < 0.01) and a tendency for reduced metastatic burden in the lungs of exercised compared to sedentary mice. The progressive marked increase in myeloid-derived suppressor cells (MDSCs) and splenomegaly seen in sedentary 4 T1 tumor-bearing mice was less pronounced in exercised mice. This difference was significant on day 17; with spleen weight (520 ± 110 mg in sedentary versus 330 ± 30 mg in exercised mice, p < 0.01) and MDSC frequency in spleen leukocytes (22.7 ± 2.6% in sedentary versus 14.3 ± 2.7% in exercised mice, p < 0.001) were significantly lower in exercised mice compared to sedentary mice. Furthermore, on day 32, the CD8+ T cell/Treg and CD8+ T cell/MDSC ratio showed a tendency to increase in tumors from exercised mice.

**Conclusions**

Our data demonstrate that exercise can slow tumor progression in a therapeutic setting. While the mechanisms of this effect require further investigation, the observed decrease in the proportion of immunosuppressive immune cells in spleen and tumor of exercised mice is likely to play a role. Importantly, the ability of exercise to reduce immunosuppression locally and systemically supports testing exercise in combination with immunotherapy as a therapeutic modality that can increase responses without increasing toxicity.

**References**

1. Ballard-Barbash R, Friedenreich CM, Courneya KS, Siddiqi SM, McTiernan A, Alfano CM: **Physical activity, biomarkers, and disease outcomes in cancer survivors: a systematic review**. *Natl Cancer Inst* 2012, **104**:815–840.

2. Koelwyn GJ, Wennerberg E, Demaria S, Jones LW: **Exercise in Regulation of Inflammation-Immune Axis Function in Cancer Initiation and Progression**. *Oncology* 2015, **29(12)**.

## Not Listed – Other

### P20 Systemic immunotherapeutic efficacy of an immunocytokine, NHS-muIL12, in a superficial murine orthotopic bladder cancer model

#### Vandeveer Amanda, John W Greiner, Jeffrey Schlom

##### Laboratory of Tumor Immunology and Biology, Center for Cancer Research, National Cancer Institute, Bethesda, MD, USA

###### **Correspondence:** Vandeveer Amanda (amanda.vandeveer@nih.gov)

**Background**

Interleukin-12 is one of the most powerful proinflammatory cytokines capable of supporting T and NK cell function, inducing IFNγ while driving a T_H_1 adaptive immune response. Its success as an antitumor agent in preclinical models has yet to be realized in a clinical setting due to systemic toxicity. An IL-12 delivery system has been developed to maximize deposition of the cytokine directly in the tumor microenvironment (TME), while mitigating the dose-limiting systemic effects.

**Methods**

NHS-IL12 is a novel immunocytokine, consisting of two molecules of human or murine IL-12 fused to a tumor necrosis-targeting human IgG1 (NHS76). NHS76 recognizes exposed chromatin-DNA found in necrotic human/murine tumors. Previous studies have shown selective tumor uptake of NHS-IL12 in necrotic subcutaneous murine tumors. Urothelial bladder cancer is known to respond favorably to immunotherapeutic agents due to many somatic mutations and TILs, and response to Bacillus Calmette-Guerin (BCG).

**Results**

We evaluated the use of NHS-muIL12 in a murine orthotopic bladder cancer model. MB49^Luc^ cells, instilled into the bladder form superficial, multifocal tumors which can be monitored with a luciferase-based intravital imaging system. NHS-muIL12 is a very potent anti-tumor agent in both MB49 tumor models, reducing tumor volume in a dose-dependent manner. In the intravesical bladder model, antitumor effects were seen at 2.5 mg/kg administrated as three separate systemic injections. Mice were cured of tumor when treated at 20 mg/kgx3 NHS-muIL12 with durable tumor-free long-term survival. Immune analyses revealed TAA-specific CTLs and IFN-γ responses, indicating the development of a specific anti-tumor immune response. An immune memory response protected mice following re-challenge with MB49 tumor cells. Anti-tumor efficacy required CD4+ or CD8+ T cells as depletion of either abrogated the anti-tumor effects. Evaluation of TILs by FACS, revealed that NHS-muIL12 significantly reduced the number of immune suppressive cells such as MDSCs, 24 hours post-treatment, which continued to the end of the study. Immunofluorescence showed correlative treatment-related modulation of CD4+ and CD8+ T cells as well as MDSCs and Tregs within the TME. Gene expression of RNA from bladder tumors, identified various immune components with immunosuppressive or immune potentiating roles, modulated by NHS-muIL12 treatment.

**Conclusions**

These data support the possibility that NHS-muIL12 abrogates an immune-suppressive response within the TME, which might permit T cells to execute their antitumor effects. NHS-huIL12 (MSB0010360N; M9241), is currently being evaluated against solid tumors in a phase I clinical trial (NCT01417546).

**Acknowledgements**

We acknowledge the kind contribution of NHS-muIL12 from EMD Serono, Billerica, MA.

## Therapeutic Cancer Vaccines

### P21 Intracellular trafficking of self-assembled immune signals

#### Michelle Bookstaver, Christopher M Jewell

##### Fischell Department of Bioengineering, University of Maryland - College Park, College Park, MD, USA

###### **Correspondence:** Michelle Bookstaver (mlbooks@umd.edu)

**Background**

We recently exploited electrostatic interaction to design self-assembling nanostructures comprised entirely from peptide antigens and toll-like receptor (TLR) agonists as adjuvants. These materials simplify vaccine composition and exhibit unique properties such as direct control over the absolute and relative concentrations of each component and co-delivery of the signals to antigen presenting cells. In pre-clinical models of melanoma, this approach leads to significantly enhanced anti-tumor immunity. Here, we study how the physicochemical features (e.g., peptide charge) and relative concentration of each component impact the internalization, trafficking, and processing of the immune signals in antigen presenting cells.

**Methods**

FITC-labeled SIINFEKL peptide was modified with three or nine arginines, for use as a cationic anchor to support self-assembly with a polyanionic nucleic acid-based TLR3 agonist, polyIC. Hollow capsules built from these signals were synthesized by coating a sacrificial CaCO_3_ core with alternating layers of modified SIINFEKL and PolyIC. After deposition, the core was removed using EDTA and capsules were washed with buffer, resulting in stable capsules formed from immune signals. Capsule size was determined by image analysis and component loading levels were determined by fluorimetry using FITC-labeled peptide and Cy5-labeled TLRa. Stability studies were carried out by incubating capsules in media as a function of different pH and ionic strengths. For uptake and trafficking studies, murine splenocytes were isolated and treated with different concentrations of capsules. Cells were analyzed by flow cytometry and imaging in the presence or absence of inhibitors of endocytic processes and during staining with markers for surface proteins and intracellular organelles.

**Results**

Capsules loaded with FITC-SIINFEKL-R_9_ and PolyIC were 1-2 μm in diameter and exhibited similar size and shape for 2 weeks when in buffer. These materials exhibited tunable loading with a composition of 15.5% peptide and 84.5% TLRa used for trafficking studies. FITC-SIINFEKL-R_9_ and PolyIC capsules were efficiently internalized through energy dependent processes (i.e., endocytosis) when incubated with primary dendritic cells within 1 hour of treatment. These effects were also found to be dose-dependent and did not impact viability of treated cells.

**Conclusions**

Initial studies reveal capsules comprised of FITC-SIIN-R_9_ and PolyIC are uptaken by primary immune cells quickly and effectively. Ongoing studies will assess the uptake of capsules by endocytosis in the presence of inhibitors to decipher the endocytic pathway and trafficking of capsules through lysosomes and endosomes.

**Acknowledgements**

This work was supported in part by NSF CAREER # 1351688 and Alliance for Cancer Gene Therapy # 15051543.

### P22 Analysis of B and T cell responses in non-small cell lung cancer (NSCLC) patients enrolled in a phase II trial of cyclophosphamide with allogenic DRibble vaccine (DPV-001)

#### Christopher Paustian^1^, Andrew Gunderson^1^, Brian Boulmay^2^, Rui Li^3^, Bradley Spieler^4^, Kyle Happel^4^, Tarsem Moudgil^5^, Zipei Feng^6^, Carmen Ballesteros-Merino^3^, Christopher Dubay^6^, Brenda Fisher^7^, Yoshinobu Koguchi^8^, Sandra Aung^1^, Eileen Mederos^4^, Carlo B. Bifulco^3^, Michael McNamara^9^, Keith Bahjat^9^, William Redmond^9^, Augusto Ochoa^4^, Hong-Ming Hu^10^, Adi Mehta^11^, Fridtjof Lund-Johansen^11^, Bernard Fox^6^, Walter Urba^8^, Rachel E. Sanborn^8^, Traci Hilton^1^

##### ^1^UbiVac, Portland, OR, USA; ^2^Section of Hematology/Oncology, Louisiana State University, New Orleans, LA, USA; ^3^Robert W. Franz Cancer Research Center, Earle A. Chiles Research Institute, Providence Cancer Center, Portland, OR, USA; ^4^Louisiana State University Stanley S. Scott Cancer Center, New Orleans, LA, USA; ^5^PPMC, Portland, OR, USA; ^6^Providence Cancer Center, Portland, OR, USA; ^7^Providence Medical Center, Portland, OR, USA; ^8^Earle A. Chiles Research Institute, Providence Cancer Center, Portland, OR, USA; ^9^Providence Medical Center, Portland, OR, USA; ^10^UbiVac, Providence Medical Center, Portland, OR, USA; ^11^Oslo University Hospital, Oslo, Norway

###### **Correspondence:** Christopher Paustian (christopher.paustian@ubivac.com)

**Background**

DRibble vaccines are microvesicles derived from proteasome-blocked autophagosomes. The DPV-001 DRibble vaccine is derived from an adenocarcinoma and a mixed histology cancer cell line. By mass spectroscopy they contain more than 130 potential NSCLC antigens, many as prospective altered-peptide ligands, which could intensify their immunogenicity. In preclinical models, DRibble immunotherapy provided significant cross-protection against 8 of 9 tumors tested. Additionally, Dribble vaccines are effective in treating established tumors in preclinical combination immunotherapy models. We hypothesize that the efficacy of DRibbles’ vaccination can be attributed to their capacity to present tumor-derived short-lived proteins (SLiPs) and defective ribosomal products (DRiPs) that are typically not processed and presented by professional antigen presenting cells. These SLiPs and DRiPs embody a prospective pool of tumor antigens against which the host may be less tolerant.

**Methods**

Thirteen definitively-treated stage III NSCLC patients were vaccinated at 3-week intervals. Patients were randomized such that some patients’ intradermal vaccines were combined with administration of imiquimod or GM-CSF as an adjuvant. PBMCs and serum were collected at baseline and at each vaccination. For one patient, PBMCs from the baseline visit and week 12 were tested against that patient’s autologous tumor cell line to measure increased tumor specific T cell activation. Studies are currently underway to evaluate changes in TCR repertoires. CD4+ and CD8+ T cells from multiple time points were sorted and TCR sequencing is being performed to look at alterations in the T cell repertoire. The primary outcome measure of this clinical trial was to discover if vaccine alone, vaccine plus imiquimod, or vaccine plus GM-CSF generated the greatest number of strong antibody response.

Serum from the baseline visit and week 12 was analyzed for increased antibody response to >9000 human proteins using ProtoArrays and Microsphere Affinity Proteomics. Where sufficient tumor was available, whole exome sequencing was done to evaluate whether antibody and T cell responses were directed to mutations, altered peptide ligands or overexpressed normal proteins.

**Results**

Compared to vaccination alone or vaccination with GM-CSF, vaccination with DPV-001 plus imiquimod significantly (p < 0.05) increased the number of antibody responses that were four-fold higher at week twelve. In the one patient where autologous tumor was available, vaccination increased the tumor-specific release of TNF-alpha by peripheral blood CD4 T cells.

**Conclusions**

Based on these studies, future trials will combine the adjuvant imiquimod with DRibble vaccine. **Trial Registration**

ClinicalTrials.gov identifier: NCT01909752

### P23 An open-label phase I/IIa escalating dose study to evaluate safety and T cell immunogenicity of PDS0101 in subjects with cervical intraepithelial neoplasia (CIN) and high-risk HPV infection

#### Frank Bedu-Addo^1^, Greg Conn^1^, Michael King^1^, Panna Dutta^1^, Robert Shepard^2^, Mark Einstein^3^

##### ^1^PDS Biotech, New Brunswick, NJ, USA; ^2^PDS Biotech, Miami Beach, FL, USA; ^3^Rutgers, NJ Medical School, Newark, NJ, USA

###### **Correspondence:** Robert Shepard (RShepard@Post.Harvard.edu)

**Background**

Current HPV vaccines are effective at preventing infection. However, there are no therapeutic vaccines to treat the infection or commonly associated diseases e.g. CIN, cervical, anal and oral cancers. A therapy that is simple, effective and safe enough to be administered to CIN and early-stage cancer patients could be important in achieving the goal of effective cancer prevention and treatment of pre-metastatic cancer. We assessed whether PDS0101, a combination of modified multi-epitope HPV16 peptides (HPVmix) and escalating doses of the synthetic Versamune® T cell activating platform could facilitate antigen cross-presentation and safe immune activation leading to strong HPV-specific CD8+ T cell induction in CIN.

**Methods**

Safety and immunogenicity were assessed in an open label dose escalation study. Groups of 3-6 subjects received either low dose (1 mg), medium dose (3 mg) or high dose (10 mg) of Versamune® cationic lipid with 2.4 mg of HPVmix. Each subject received one SC dose every 3 weeks for a total of 3 doses. T cell response was evaluated by IFN-γ and granzyme-b ELISpot using blood drawn from the subjects pre-vaccination, 2 weeks after each vaccination and 90 days after vaccination 3. The trial is registered at ClinicalTrials.gov (number NCT02065973).

**Results**

No serious adverse events were reported. No IND safety reports were submitted. No subjects withdrew. Strong HPV-specific T cell responses occurred at all 3 doses, even in those subjects with low pre-vaccination T cell responses. PDS0101 vaccination led to strong T cell responses evaluated by both IFN-γ and granzyme-b ELISpot. **Conclusions:** PDS0101 is safe and effectively performs antigen cross-presentation as demonstrated by HPV-specific T cell responses, including inducing active cytolytic T cells. Clinical benefit in CIN2/3 and cancer will be evaluated in larger phase II trials.

**Conclusions**

PDS0101 is safe and effectively performs antigen cross-presentation as demonstrated by HPV-specific T cell responses, including inducing active cytolytic T cells. Clinical benefit in CIN2/3 and cancer will be evaluated in larger phase II trials.

**Trial Registration**

ClinicalTrials.gov identifier: NCT02065973

## Tumor Microenvironment

### P24 Effects of TLR7 agonist imiquimod combined with local radiotherapy on the tumor microenvironment in women with metastatic breast cancer in a prospective trial

#### Sylvia Adams^1^, Ena Wang^2^, Ping Jin^3^, Yelena Novik^1^, Debra Morrison^1^, Ruth Oratz^1^, Franco M Marincola^2^, David Stroncek^4^, Judith Goldberg^1^, Sandra Demaria^5^, Silvia C Formenti^5^

##### ^1^Perlmutter Cancer Center, New York University School of Medicine, NYC, NY, USA; ^2^Sidra Medical and Research Center, Doha, Qatar; ^3^National Institutes of Health Clinical Center Department of Transfusion Medicine, Bethesda, MD, USA; ^4^National Institutes of Health Clinical Center, Bethesda, MD, USA; ^5^Weill Cornell Medicine, Department of Radiation Oncology, New York, NY, USA

#### **Correspondence:** Sylvia Adams (sylvia.adams@nyumc.org)

**Background**

Application of TLR7 activator imiquimod (IMQ) onto BCC of the skin leads to an early tumoral transcriptional profile of immunological rejection (ICR) preceding complete remission as shown in a randomized trial [1]. Here we employed the same methodology evaluating serial FNA tumor biopsies from breast cancer patients treated on a clinical trial of the combination of IMQ and radiotherapy (RT), to delineate dynamic changes associated with ICR in breast cancer and to understand the contribution of each treatment modality to antitumor immunity *in vivo*. We previously demonstrated synergy of IMQ/RT in the poorly immunogenic TSA model with enhanced T cell-mediated inhibition of treated and untreated tumors [2].

**Methods**

Clinical trial (NCT01421017): for patients with metastatic breast cancer to the skin. Treatment: topical IMQ to one metastasis, IMQ and RT to another metastasis. IMQ self-applied 5xX/per week x 8 weeks, RT started with first IMQ (6Gyx5 over 10 days). Cyclophosphamide (200 mg/m2 IV) was administered a week before in a subset of patients. An untreated, measurable lesion (skin or distant metastases) outside the IMQ and radiation fields was observed as systemic response read-out per RECIST1.1. Local response defined as PR or CR in treated lesions. FNA of IMQ and IMQ/RT treated metastases: at baseline, 2 and 3 weeks, RNA isolation/amplification performed per SOPs. Gene expression: Affymetrix Human GeneArray 1.0 ST/Partek Genomics suite software with special emphasis on expected immune signature.

**Results**

Serial FNA samples are available from 18 patients. Gene expression profiles of baseline biopsy treated with IMQ/RT identified 2309 differentially expressed genes (p < 0.005) between CR and PR. Among them, ICR genes such as GZMB, GZMH, PRF1, GNLY, CD8A and TBX21 are over expressed in CR. Significant altered gene expression was observed in progressing lesions (week 3 vs baseline, 1854 genes) in contrast to responding metastases (PR: 53 genes, CR: 23 genes) post IMQ/RT suggesting active wound healing and tumor progression signature. For the IMQ alone treated metastases, differential gene expression was observed at baseline distinguishing subsequent PR and PD (n = 189, p < 0.005). Systemic response was observed as a marked baseline gene expression difference (n = 1177, p < 0.005) predicting abscopal phenomena (CR, PR, SD and PD).

**Conclusions**

The ICR signature in tumors before IMQ-RT treatment is positively correlated with complete local response, which validates the ICR hypothesis in metastatic breast cancer. Systemic response consistent with induction and/or boosting of adaptive immunity is predicted by significant enrichment of immune signature.

**Acknowledgements**

1RO1CA161891

**Trial Registration**

ClinicalTrials.gov identifier: NCT01421017

**References**

1. Panelli: *Genome Biol* 2008.

2. Dewan: *CCR* 2012.

### P25 Immunoscore as a prognostic marker in stage I-III colon cancer: results of a SITC-led global validation study

#### Jérôme Galon^1^, Bernhard Mlecnik^1^, Florence Marliot^1^, Fang-Shu Ou^2^, Carlo B Bifulco^3^, Alessandro Lugli^4^, Inti Zlobec^4^, Tilman T Rau^4^, Iris D Nagtegaal^5^, Elisa Vink-Borger^5^, Arndt Hartmann^6^, Carol Geppert^6^, Michael H. Roehrl^7^, Prashant Bavi^7^, Pamela S Ohashi^7^, Julia Y Wang^7^, Linh T Nguyen^7^, SeongJun Han^7^, Heather L MacGregor^7^, Sara Hafezi-Bakhtiari^7^, Bradley G Wouters^7^, Yutaka Kawakami^8^, Boryana Papivanova^8^, Mingli Xu^8^, Tomonobu Fujita^8^, Shoichi Hazama^9^, Nobuaki Suzuki^9^, Hiroaki Nagano^9^, Kiyotaka Okuno^10^, Kyogo Itoh^11^, Eva Zavadova^12^, Michal Vocka^12^, Jan Spacek^12^, Lubos Petruzelka^12^, Bohuslav Konopasek^12^, Pavel Dundr^12^, Helena Skalova^12^, Toshihiko Torigoe^13^, Noriyuki Sato^13^, Tomohisa Furuhata^13^, Ichiro Takemasa^13^, Marc Van den Eynde^14^, Anne Jouret-Mourin^14^, Jean-Pascal Machiels^14^, Tessa Fredriksen^1^, Lucie Lafontaine^1^, Bénédicte Buttard^1^, Sarah Church^1^, Pauline Maby^1^, Helen Angell^1^, Mihaela Angelova^1^, Angela Vasaturo^1^, Gabriela Bindea^1^, Anne Berger^1^, Christine Lagorce^1^, Prabhu S Patel^15^, Hemangini H Vora^15^, Birva Shah^15^, Jayendrakumar B Patel^15^, Kruti N Rajvik^15^, Shashank J Pandya^15^, Shilin N Shukla^15^, Yili Wang^16^, Guanjun Zhang^16^, Giuseppe V Masucci^17^, Emilia K Andersson^17^, Fabio Grizzi^18^, Luigi Laghi^18^ Gerardo Botti^19^, Fabiana Tatangelo^19^, Paolo Delrio^19^, Gennaro Cilberto^19^, Paolo A Ascierto^19^, Franco Marincola^20^, Daniel J Sargent^2^, Bernard A Fox^3^, Franck Pages^1^

##### ^1^INSERM, Université Pierre et Marie Curie, Université Paris Descartes, Paris, France; ^2^Mayo Clinic, Rochester, MN, USA; ^3^Earle A. Chiles Research Institute, Providence Cancer Center, Portland, Oregon, USA; ^4^Institute of Pathology, University of Bern, Bern, Switzerland; ^5^Radboud University Nijmegen Medical Center, Nijmegen, Netherlands; ^6^University Erlangen-Nürnberg, Erlangen, Germany; ^7^Princess Margaret Cancer Centre, University Health Network, Toronto, ON, Canada; ^8^Division of Cellular Signaling, Institute for Advanced Medical Research, Keio University School of Medicine, Tokyo, Japan; ^9^Department of Gastroenterological, Breast and Endocrine Surgery, Yamaguchi University Graduate School of Medicine, Ube, Japan; ^10^Department of Surgery, Kinki University Faculty of Medicine, Osaka-Sayama, Japan; ^11^Division of Clinical Research, Research Center for Innovative Cancer Therapy, Kurume University School of Medicine, Kurume, Japan; ^12^First Faculty of Medicine, Charles University and General University Hospital, Prague, Czech Republic; ^13^Sapporo Medical University, Sapporo, Japan; ^14^Institut Roi Albert II, Cliniques universitaires St-Luc, Université Catholique de Louvain, Brussels, Belgium; ^15^The Gujarat Cancer & Research Institute, Ahmedabad, India; ^16^Institute for Cancer Research, Xi’an Jiaotong University, Xi’an, China; ^17^Karolinska Institutet, Karolinska University, Stockholm, Sweden; ^18^Humanitas Clinical and Research Center, Rozzano, Italy; ^19^Istituto Nazionale Tumori Fondazione Pascale, Naples, Italy; ^20^Sidra Medical and Research Center, Doha, Qatar

###### **Correspondence:** Bernard A Fox (bernard.fox@providence.org)

**Background**

Increasing evidence has illustrated that enhanced lymphocytic infiltration is a powerful prognostic marker in colon cancer (CC). The Immunoscore (IM) methodology was developed as a standardized assay to quantify the *in situ* immune cell infiltrate.

**Methods**

The Society for Immunotherapy of Cancer (SITC) led an international consortium, initiated with 23 expert centers from 17 countries, to evaluate the Immunoscore in routine clinical settings. CC patients (pts) stages I/II/III with no prior neo-adjuvant treatment were included in this study. Overall, 3855 pts split into a training set (TS), internal validation set (IVS), and external validation set (EVS) were quantified for IM using immunohistochemistry with CD3/CD8 antibodies and digital pathology quantification of whole slide sections. All statistical analyses were pre-defined and performed by external statisticians. The primary endpoint was time-to-recurrence (TTR); multivariate analyses were performed using Cox models adjusted for IM, age, gender, T-stage, N-stage, and stratified by participating center.

**Results**

Across centers, the median recurrent follow-up was 126.6 months. Pt characteristics: 51.5% male, median age 69 years, and 17%/54%/29% stage I/II/III, respectively. Among pts with stages I-III CC in the TS, TTR was shorter among 152 pts (22%) with Low-IM CC vs. 548 pts with High-IM CC (HR [95% CI], 0.41 [0.28-0.61]; P < 0.0001). In the IVS, TTR was also shorter among 155 pts with Low-IM CC vs. 481 pts with High-IM CC (0.41 [0.27-0.65]; P < 0.0001). In the EVS, TTR was also shorter among 225 pts with Low-IM CC vs. 744 pts with High-IM CC (0.51 [0.38-0.68]; P < 0.0001). These results were independent of age, sex, tumor stage, and sidedness. Among secondary objectives, Immunoscore groups (High, Int, Low) predicted time to recurrence in the TS (0.19 [0.10-0.37]; P < 0.0001), IVS (0.27 [0.14-0.53]; P < 0.0001), and EVS (0.33 [0.22-0.49]; P < 0.0001). In stage II CC pts (1433), the difference in TTR was significant between the Low and High-Immunoscore groups (0.36 [0.23-0.56]; P < 0.0001). In multivariate models, Immunoscore grouping (2, 3, or 5) was significant (C-index : 0.73 [0.66-0.80], all P < 0.0001). Multivariate models including MSI and sidedness were performed and will also be presented. Reproducibility of the IM assay was validated across centers.

**Conclusions**

The primary and secondary endpoints of the global Immunoscore study were reached. Overall, TTR was significantly longer in pts with stages I-III CC defined as High-IM. Moreover, a subgroup of patients with high-risk stage II CC was also identified by Low-IM.

**Acknowledgements**

This initiative was supported by a variety of sources, including funding from Definiens, Prometheus, and a grant from the Czech Ministry of Health, 15-28188A and League against cancer.

### P26 Defining critical features of the immune microenvironment in melanoma

#### Robyn Gartrell^1^, Douglas Marks^1^, Edward Stack^2^, Yan Lu^1^, Daisuke Izaki^3^, Kristen Beck^4^, Dan Tong Jia^4^, Paul Armenta^4^, Ashley White-Stern^4^, Yichun Fu^4^, Zoe Blake^1^, Howard L Kaufman^5^, Bret Taback^1^, Basil Horst^1^, Yvonne M Saenger^6^

##### ^1^Columbia University Medical Center, New York, NY, USA; ^2^Perkin Elmer, Hopkinton, MA, USA; ^3^Columbia University, New York, NY, USA; ^4^Columbia University College of Physicians and Surgeons, New York, NY, USA; ^5^Rutgers Cancer Institute of New Jersey, New Brunswick, NJ, USA; ^6^New York Presbyterian/Columbia University Medical Center, New York, NY, USA

###### **Correspondence:** Robyn Gartrell (rdg2129@columbia.edu)

**Background**

Precise biomarkers are urgently needed to characterize the tumor immune micro-environment, both for prognostication and to predict the benefit of immuno-therapeutic intervention. HLA-DR on tumor cells and Ki67 on cytotoxic (CD8+) T cells have been proposed as biomarkers of anti-PD1 activity. Multiplex immunohistochemistry (mIHC) allows for automated quantitation of phenotypes and spatial distributions of immune cell populations within formalin fixed paraffin embedded (FFPE) tissues.

**Methods**

**Fig. 9 (abstract P26).**
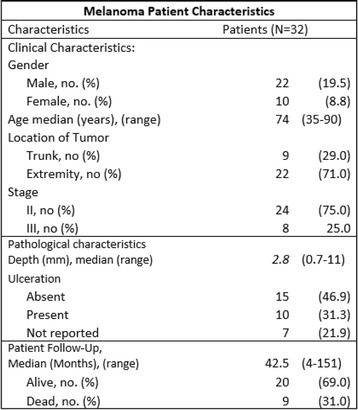
Demographic Characteristics of Melanoma

**Fig. 10 (abstract P26).**
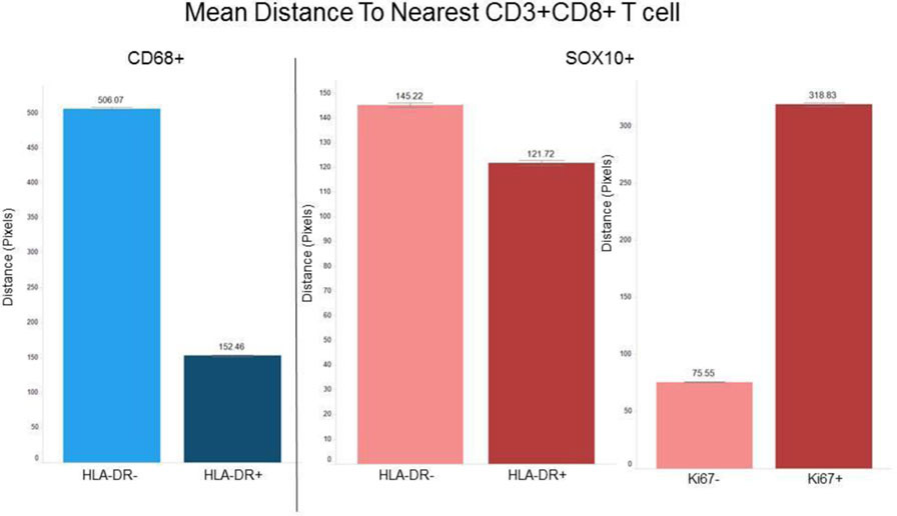
Distance between CD3 + CD8+ T cells and nearest neighbor, Left: Mean distance from CD3 + CD8+ to CD68 + HLA-DR- (light blue) or CD68 + HLA-DR+ (dark blue), Center: Mean distance from CD3 + CD8+ to SOX10 + HLA-DR- (pink) or SOX10 + HLA-DR+ red), Right: Mean distance from CD3 + CD8+ to SOX10 + Ki67- (pink) or SOX10 + Ki67+ red)

**Fig. 11 (abstract P26).**
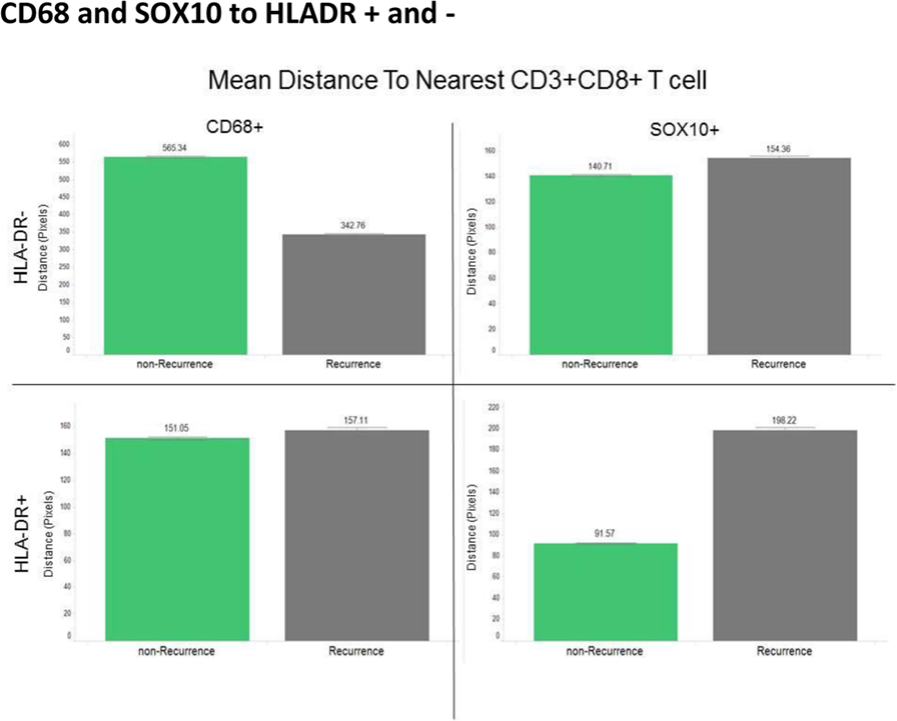
Distance between CD3 + CD8+ T cells and nearest neighbor be recurrence status, Top Left: Mean distance from CD3 + CD8+ to CD68 + HLA-DR- by recurrence status, Lower Left: Mean distance from CD3 + CD8+ to CD68 + HLA-DR+ by recurrence status. Top Right: Mean distance from CD3 + CD8+ to SOX10 + HLA-DR- by recurrence status, Bottom Right: Mean distance from CD3 + CD8+ to SOX10 + HLA-DR+ by recurrence status

In order to test whether mIHC can better characterize the tumor immune microenvironment, we screened databases at the Herbert Irving Cancer Center (HICC) at Columbia University for early stage melanoma patients with available FFPE primary melanoma tissue and documented clinical follow up. We identified a preliminary population of 31 stage II-III melanoma patients diagnosed between 2000 and 2012, with characteristics shown in Fig. 9 for whom pathology from the primary biopsy was shown. Clinical follow up was available on 18 patients of whom 9 patients were alive with no evidence of recurrence, 1 had died of another malignancy, and 7 had died of melanoma. 15 patients had more than 24 months of survival information available but no detailed clinical information. 5 μm slides from either the primary biopsy or subsequent wide local excision procedure were stained using Opal multiplex IHC for DAPI, CD3 (LN10, Leica), CD8 (4B11, Leica), CD68 (KP1, Biogenex), SOX10 (BC34, Biocare), HLA-DR (LN-3, Abcam) and Ki67 (MIB1, Abcam). Cell phenotypes within representative fields pre-selected by a trained dermato-pathologist and were visualized using the Mantra quantitative pathology workstation (Perkin Elmer), and analysis of spatial distribution of CD3 + CD8+ cells analyzed as shown in Figs. 10 and 11 using inForm® image analysis software (Perkin Elmer), and Spotfire software (TIBCO).

**Results**

CD3 + CD8+ cells are closer to both tumor (SOX10+) and CD68+ cells when they express HLA-DR (p < 0.001). Conversely, CD3 + CD8+ cells are significantly farther from Sox10+ cells when they express Ki-67. Among patients with clinical follow up, CD3 + CD8+ cells in non-recurrent patients were closer to SOX10+HLA-DR+  cells than they were in recurrent patients(p < 0.001).

**Conclusions**

If proximity is a surrogate for interaction, these data may indicate that HLA-DR expression enhances interaction with T cells for both CD68+ infiltrating cells and Sox10+ tumor cells. In addition, CD3+CD8+ cells were closer to SOX10+HLA-DR+  cells in patients who did not recur, which is interesting in light of recent data showing that expression of HLA-DR by tumor cells increases likelihood of response to anti-PD1. Further staining and analysis of annotated tumor samples from the complete HICCC cohort 2000-2012 is ongoing and results will be updated at time of presentation.

### P27 Immune profiling via multiplexed immunofluorescence shows that Imprime based anti-cancer efficacy involves profound changes in macrophage polarization, type 1 IFN signaling and the formation of immune cell clusters

#### Steven Leonardo, Keith Gorden, Ross B Fulton, Kathryn Fraser, Takashi O Kangas, Richard Walsh, Kathleen Ertelt, Jeremy Graff, Mark Uhlik

##### Biothera Pharmaceuticals Inc., Eagan, MN, USA

###### **Correspondence:** Steven Leonardo (sleonardo@biothera.com)

**Background**

Imprime PGG (Imprime) is a soluble, intravenously (iv) administered yeast 1,3/1,6 β-glucan PAMP (pathogen-associated molecular pattern). As a PAMP, Imprime triggers innate immune function, including direct tumor killing, repolarization of the immunosuppressive tumor microenvironment (flipping immunosuppressive M2 macrophages to an anti-tumor M1 state), and T cell expansion and activation via dendritic cell maturation and antigen presentation. Clinically, Imprime has demonstrated promising efficacy in clinical trials when combined with tumor-targeting or anti-angiogenic antibodies. Phase II studies with pembrolizumab are starting in both metastatic triple negative breast cancer and metastatic melanoma. Herein, we have employed multiplexed immunofluorescence to profile the immune microenvironment in preclinical tumor tissues.

**Methods**

The B16F10 experimental metastasis model was used to interrogate Imprime’s anti-tumor activity *in vivo*. B16F10 melanoma cells were injected into the tail vein of syngeneic C57BL/6 mice, seeding the lungs with B16 foci. Outgrowth of these metastatic foci was assessed after treatment with the tumor-targeting anti-tryp1 antibody TA99, Imprime, or the combination. At various times post tumor injection, lungs were examined via multiplexed immunofluorescence (IFC) for markers of immune infiltration and activation. IFC was performed using 7-color staining (Opal technology, PerkinElmer) combined with *in situ* hybridization (RNAScope, ACD). Images were acquired with the Vectra3 multispectral imaging system and cells segmented using Inform (PerkinElmer). Imaging data were transformed into “.fcs” files and analyzed using Flowjo flow cytometry software (Treestar)). Relational parameters such as immune cell clustering and tumor infiltration were performed via custom algorithms in R.

**Results**

TA99 alone suppressed the outgrowth of B16 lung metastases by 54% when compared to vehicle treatment. The combination of Imprime with TA99 reduced the number of metastases even more profoundly (96% vs vehicle). IFC analyses showed that Imprime specifically accumulates in the tumor stroma, binds to macrophages and elicits increased iNOS production, indicating the re-polarization of these macrophages to a more M1-like, inflammatory state. Imprime-treatment also triggered the formation of large immune cell clusters, possibly representing resolved tumor nests or the establishment of tertiary lymphoid tissues, both of which have been identified as predictors of successful anti-tumor immune responses. Finally, Imprime treatment and localization at the tumor site corresponds with substantial upregulation of the gene Mx1- a type 1 interferon-responsive gene.

**Conclusions**

Imprime is a potent immunomodulator that induces a coordinated immune attack *in vivo* demonstrated by immune cell binding, M1 re-polarization and a type-1 interferon signature that coincides with reduced outgrowth of established ling metastases.

### P28 Local convection-enhanced delivery of PD-1 blockade antibody in de novo murine model of glioblastoma

#### Jennifer S Sims, Liang Lei, Takashi Tsujiuchi, Jeffrey N Bruce, Peter Canoll

##### Columbia University Medical Center, New York, NY, USA

###### **Correspondence:** Jennifer S Sims (jennifer.s.sims@gmail.com)

**Background**

Systemic delivery of anti-PD1 antibody therapy has proven relatively safe in glioma patients, but therapeutic response remains unpredictable and persistently low. Checkpoint blockade antibodies face numerous potential confounders in these tumors, such as the blood-brain barrier, poor local or lymph node presentation of tumor antigens, and unknown dependency on PD-1/PD-L1 activity during tumor progression. Here, we conducted a pilot study using intracranial convection-enhanced delivery (CED) of anti-PD1 (mDX400) into a *de novo* murine glioma model to the dissect tumor and immune perturbations following local treatment, and to compare the efficacy of treating during early or late tumor progression.

**Methods**

Transgenic C57BL/6-PTEN(fl/fl) mice were injected with a retrovirus expressing PDGFb and cre recombinase, inducing tumorigenesis as previously described. In this model, with a median survival of 80 days post-tumor induction (D80), convergence to a stereotyped subset of genomic rearrangements occurs by approximately D35. Intracranial osmotic pumps filled with mDX400 or isotype control antibody solution were implanted at the tumor site for 14-day windows spanning (D28-D42) or following (D42-D56) this developmental transition, then removed. Tumor burden was monitored by bioluminescence (luciferase reporter), and mice were sacrificed upon presentation of tumor-related morbidity. Tissue was formalin-fixed for histopathology and cryopreserved for gene expression analysis.

**Results**

During both treatment windows, tumor burden decreased differentially in mDX400-treated mice. While survival time between mDX400- and isotype-treated mice was nearly identical for D28-D42 (both median D70), for mice treated between D42-D56, median survival differed (D88 vs. D68), but without statistical significance between the groups (p = 0.25). Interestingly, the D42-D56 mDX400 group produced several “long-term survivors”, who lived up to 158 days with stable tumor burden. While substantial T cell infiltration was detected in the end-stage tumors of both mDX400- and isotype-treated mice by immunohistochemistry (CD3e), expression of immune signaling pathways (e.g., Fc receptor and Toll-like receptor families, phagosome/lysosome components), was significantly higher among three long-surviving mDX400-treated mice than in three isotype-treated mice.

**Conclusions**

Our pilot study of mDX400 administration by CED identified an impact on tumor burden during and following therapy, but a lack of survival benefit for D28-D42 treatment. While additional experiments are needed to statistically evaluate survival benefit for the later treatment window, differentially high intratumoral expression of genes reflecting immune activation among mDX400-treated, long-surviving mice demonstrates that molecular study in this model may elucidate intratumoral conditions associated with response to anti-PD1 blockade in glioma.

**Acknowledgements**

This pre-clinical study is supported by Merck & Co. Investigator-Initiated Sponsored Projects grant LKR146174.

